# Supplementary material for: Data-Driven Radiogenomic Approach for Deciphering Molecular Mechanisms Underlying Imaging Phenotypes in Lung Adenocarcinoma: A Pilot Study
Source: Int J Mol Sci. 2023 Mar 3;24(5):4947. doi: 10.3390/ijms24054947 (PMC10003564; doi:10.3390/ijms24054947)
Supplement: Supplementary file 1 [file ijms-24-04947-s001.zip › Supplement/ALL_Tables_Supplementary Material Final 13.02.23.pdf]

Supplementary information

Data-Driven Radiogenomic Approach for Deciphering Molecular Mechanisms Underlying Imaging Phenotypes in Lung Adenocarcinoma: A Pilot Study

Table S1: List of 317 significant GO terms for DEGs with the associated number of significant radiomic associations as resulted from piano analysis.

Pathway	Number of significant radiomic associations
nuclear division	63
meiotic cell cycle	59
meiotic cell cycle process	57
meiotic nuclear division	55
external encapsulating structure organization	51
extracellular structure organization	51
embryonic skeletal system morphogenesis	50
extracellular matrix organization	50
skeletal system development	50
skeletal system morphogenesis	50
animal organ morphogenesis	49
central nervous system neuron differentiation	49
biological adhesion	48
cardiac chamber development	48
cardiac ventricle development	48
cell adhesion	48
collagen metabolic process	48
embryo development	48
urogenital system development	48
anatomical structure formation involved in morphogenesis	47
appendage development	47
circulatory system development	47
head development	47
limb development	47
mesenchyme development	47
regulation of anatomical structure morphogenesis	47
anatomical structure morphogenesis	46
brain development	46
kidney development	46
regulation of developmental process	46
regulation of multicellular organismal process	46
tube development	46
animal organ development	45

developmental process	45
embryonic morphogenesis	45
embryonic skeletal system development	45
heart development	45
regulation of nervous system development	45
system development	45
tube morphogenesis	45
ureteric bud development	45
anatomical structure development	44
blood vessel development	44
central nervous system development	44
homophilic cell adhesion via plasma membrane adhesion molecules	44
morphogenesis of a branching epithelium	44
multicellular organism development	44
multicellular organismal process	44
neuron migration	44
positive regulation of developmental process	44
regulation of multicellular organismal development	44
renal system development	44
tissue morphogenesis	44
vasculature development	44
blood vessel morphogenesis	43
branching morphogenesis of an epithelial tube	43
cardiac chamber morphogenesis	43
cell differentiation	43
cell junction organization	43
cell morphogenesis	43
cellular developmental process	43
digestive tract development	43
morphogenesis of a branching structure	43
negative regulation of developmental process	43
pattern specification process	43
tissue development	43
cell part morphogenesis	42
cell projection morphogenesis	42
embryonic organ development	42
embryonic organ morphogenesis	42
mesonephros development	42
nervous system development	42
plasma membrane bounded cell projection morphogenesis	42
regionalization	42
regulation of localization	42
angiogenesis	41
axon development	41
axonogenesis	41
cell-cell adhesion via plasma-membrane adhesion molecules	41
forebrain development	41

regulation of cell adhesion	41
synapse organization	41
cell communication	40
cell migration	40
neurogenesis	40
regulation of cell differentiation	40
signaling	40
cell development	39
cellular component morphogenesis	39
neuron projection morphogenesis	39
cell fate specification	38
cellular response to organic substance	38
extracellular matrix disassembly	38
negative regulation of multicellular organismal process	38
positive regulation of multicellular organismal process	38
regulation of cell development	38
regulation of cellular component movement	38
regulation of signaling	38
cardiac ventricle morphogenesis	37
cell junction assembly	37
cellular response to stimulus	37
kidney epithelium development	37
neural precursor cell proliferation	37
generation of neurons	36
ossification	36
response to stimulus	36
cell surface receptor signaling pathway	35
epithelium development	35
regulation of locomotion	35
telencephalon development	35
cell population proliferation	34
cell-cell adhesion	34
cell morphogenesis involved in differentiation	33
cell motility	33
localization of cell	33
locomotion	33
cell fate commitment	32
neuron differentiation	32
positive regulation of cell differentiation	32
positive regulation of nervous system development	32
regulation of synapse assembly	32
response to endogenous stimulus	32
response to organic substance	32
carboxylic acid transport	31
cell morphogenesis involved in neuron differentiation	31
neuron fate commitment	31
actin filament-based process	30

neuron projection development	30
response to wounding	30
cytoskeleton organization	29
epithelial cell differentiation	29
neuron development	29
regulation of biological quality	29
developmental process involved in reproduction	28
neuron projection guidance	28
regulation of wound healing	28
cell differentiation in spinal cord	27
diencephalon development	27
positive regulation of cell population proliferation	27
regulation of ERK1 and ERK2 cascade	27
response to chemical	27
wound healing	27
cell-cell signaling	26
cellular response to chemical stimulus	26
chemotaxis	26
neuropeptide signaling pathway	26
sensory organ development	26
sensory organ morphogenesis	26
sensory perception of mechanical stimulus	26
taxis	26
circulatory system process	24
ERK1 and ERK2 cascade	24
negative regulation of wound healing	24
positive regulation of secretion by cell	24
reproduction	24
reproductive process	24
cornification	23
localization	23
movement of cell or subcellular component	23
negative regulation of response to wounding	23
positive regulation of ERK1 and ERK2 cascade	23
axon guidance	22
muscle structure development	22
signal release	22
blood circulation	21
regulation of cell population proliferation	21
regulation of ossification	21
muscle tissue development	20
negative regulation of blood coagulation	20
positive regulation of secretion	20
system process	20
homeostatic process	19
regulation of secretion	18
regulation of transport	18
vascular process in circulatory system	18
calcium ion homeostasis	17

negative regulation of hemostasis	17
organic anion transport	17
positive regulation of MAPK cascade	17
regulation of secretion by cell	17
response to external stimulus	17
secretion	17
transmission of nerve impulse	17
action potential	16
biomineral tissue development	16
lipid transport	16
neutrophil chemotaxis	16
organic hydroxy compound transport	16
regulation of ion transport	16
striated muscle tissue development	16
cellular calcium ion homeostasis	15
export from cell	15
monocarboxylic acid transport	15
multicellular organismal signaling	15
organic acid transport	15
regulation of body fluid levels	15
regulation of system process	15
response to lipid	15
response to oxygen-containing compound	15
chemical homeostasis	14
positive regulation of calcium ion transport	14
response to corticosteroid	14
retinoid metabolic process	14
secretion by cell	14
cell-cell junction organization	13
divalent inorganic cation homeostasis	13
excretion	13
heart process	13
regulation of blood circulation	13
regulation of blood pressure	12
sensory perception of pain	12
cellular divalent inorganic cation homeostasis	11
sodium ion transmembrane transport	11
sodium ion transport	11
adenylate cyclase-activating G protein-coupled receptor signaling pathway	10
antimicrobial humoral response	10
bone remodeling	10
calcium ion transport	10
digestion	10
drug metabolic process	10
endocrine process	10
multicellular organismal reproductive process	10
regulation of calcium ion transport	10
regulation of cation transmembrane transport	10
regulation of heart contraction	10
regulation of transporter activity	10

response to hormone	10
digestive system process	9
multicellular organism reproduction	9
phospholipase C-activating G protein-coupled receptor signaling pathway	9
regulation of metal ion transport	9
terpenoid metabolic process	9
cellular metal ion homeostasis	8
heart contraction	8
muscle organ development	8
nervous system process	8
regulation of renal system process	8
response to xenobiotic stimulus	8
sexual reproduction	8
amine transport	7
behavior	7
cation homeostasis	7
cellular cation homeostasis	7
cellular chemical homeostasis	7
cognition	7
icosanoid metabolic process	7
metal ion homeostasis	7
metal ion transport	7
multicellular organismal homeostasis	7
positive regulation of cytosolic calcium ion concentration	7
positive regulation of transport	7
regulation of ion transmembrane transport	7
regulation of transmembrane transport	7
tissue homeostasis	7
adenylate cyclase-modulating G protein-coupled receptor signaling pathway	6
learning or memory	6
multi-organism process	6
multi-organism reproductive process	6
positive regulation of icosanoid secretion	6
regulation of amine transport	6
regulation of cytosolic calcium ion concentration	6
regulation of membrane potential	6
regulation of systemic arterial blood pressure	6
cellular ion homeostasis	5
icosanoid transport	5
inorganic ion homeostasis	5
ion homeostasis	5
modulation of chemical synaptic transmission	5
muscle contraction	5
muscle system process	5
regulation of hormone levels	5
response to ketone	5
anterograde trans-synaptic signaling	4

cation transmembrane transport	4
cation transport	4
cellular homeostasis	4
cellular response to xenobiotic stimulus	4
chemical synaptic transmission	4
inorganic cation import across plasma membrane	4
inorganic ion import across plasma membrane	4
negative regulation of endopeptidase activity	4
negative regulation of peptidase activity	4
positive regulation of prostaglandin secretion	4
potassium ion transmembrane transport	4
potassium ion transport	4
regulation of bone remodeling	4
regulation of trans-synaptic signaling	4
response to mechanical stimulus	4
response to organic cyclic compound	4
response to organophosphorus	4
trans-synaptic signaling	4
transmembrane transport	4
anatomical structure maturation	3
developmental maturation	3
neuron fate specification	3
positive regulation of protein tyrosine kinase activity	3
response to cAMP	3
synaptic signaling	3
actin-mediated cell contraction	2
catecholamine secretion	2
feeding behavior	2
inorganic cation transmembrane transport	2
inorganic ion transmembrane transport	2
ion transmembrane transport	2
lipid export from cell	2
regulation of catecholamine secretion	2

Table S2: List of 538 significant GO terms for DEMs with the associated number of significant radiomic associations as resulted from piano analysis.

Pathway	Number of significant radiomic associations
cellular response to interleukin-6	31
interleukin-6-mediated signaling pathway	31
negative regulation of interleukin-6-mediated signaling pathway	31
regulation of interleukin-6-mediated signaling pathway	31

response to interleukin-6	31
negative regulation of cytokine-mediated signaling pathway	20
regulation of cytokine-mediated signaling pathway	20
negative regulation of response to cytokine stimulus	19
regulation of response to cytokine stimulus	19
response to organic substance	18
cellular response to organic substance	16
cellular response to chemical stimulus	15
cellular response to transforming growth factor beta stimulus	13
positive regulation of cell migration involved in sprouting angiogenesis	13
response to transforming growth factor beta	13
transforming growth factor beta receptor signaling pathway	13
transmembrane receptor protein serine/threonine kinase signaling pathway	10
negative regulation of cell cycle G1/S phase transition	9
negative regulation of cell cycle process	9
negative regulation of peptide secretion	9
negative regulation of protein secretion	9
negative regulation of secretion by cell	9
positive regulation of apoptotic process	9
positive regulation of cell death	9
positive regulation of programmed cell death	9
negative regulation of protein kinase B signaling	8
negative regulation of transmembrane receptor protein serine/threonine kinase signaling pathway	8
regulation of MAPK cascade	8
cell cycle G1/S phase transition	7
negative regulation of cell cycle	7
negative regulation of cell cycle phase transition	7
negative regulation of G1/S transition of mitotic cell cycle	7
negative regulation of muscle cell apoptotic process	7
regulation of cell communication	7
regulation of cell cycle G1/S phase transition	7
regulation of signaling	7
regulation of transmembrane receptor protein serine/threonine kinase signaling pathway	7
amide biosynthetic process	6
miRNA mediated inhibition of translation	6
muscle structure development	6
negative regulation of mitotic cell cycle phase	6

transition	
negative regulation of secretion	6
negative regulation of translation	6
negative regulation of translation, ncRNA-mediated	6
peptide biosynthetic process	6
regulation of cellular macromolecule biosynthetic process	6
regulation of macromolecule biosynthetic process	6
regulation of translation	6
regulation of translation, ncRNA-mediated	6
translation	6
cell cycle phase transition	5
cellular amide metabolic process	5
G1/S transition of mitotic cell cycle	5
matrix metallopeptidase secretion	5
muscle cell differentiation	5
muscle cell proliferation	5
negative regulation of cellular amide metabolic process	5
negative regulation of matrix metallopeptidase secretion	5
negative regulation of transporter activity	5
peptide metabolic process	5
positive regulation of cellular process	5
positive regulation of endothelial cell apoptotic process	5
regulation of cell cycle phase transition	5
regulation of cell cycle process	5
regulation of cellular amide metabolic process	5
regulation of G1/S transition of mitotic cell cycle	5
regulation of matrix metallopeptidase secretion	5
regulation of response to stress	5
regulation of signal transduction	5
cardiac muscle cell membrane repolarization	4
epithelial cell apoptotic process	4
intracellular signal transduction	4
membrane repolarization	4
membrane repolarization during action potential	4
membrane repolarization during cardiac muscle cell action potential	4
negative regulation of calcium ion transmembrane transport	4
negative regulation of cardiac muscle cell apoptotic process	4
negative regulation of cellular macromolecule biosynthetic process	4
negative regulation of cellular protein	4

metabolic process	
negative regulation of DNA-binding transcription factor activity	4
negative regulation of macromolecule biosynthetic process	4
negative regulation of MAPK cascade	4
negative regulation of membrane repolarization during cardiac muscle cell action potential	4
negative regulation of response to stimulus	4
negative regulation of smooth muscle cell proliferation	4
negative regulation of striated muscle cell apoptotic process	4
positive regulation of biological process	4
positive regulation of cell fate commitment	4
positive regulation of epithelial cell apoptotic process	4
positive regulation of muscle cell differentiation	4
regulation of cardiac muscle cell membrane repolarization	4
regulation of cell fate commitment	4
regulation of DNA-binding transcription factor activity	4
regulation of epithelial cell apoptotic process	4
regulation of membrane repolarization	4
regulation of membrane repolarization during action potential	4
regulation of membrane repolarization during cardiac muscle cell action potential	4
regulation of muscle cell differentiation	4
regulation of response to stimulus	4
regulation of smooth muscle cell proliferation	4
smooth muscle cell proliferation	4
apoptotic process	3
cardiac muscle cell apoptotic process	3
cardiocyte differentiation	3
cell death	3
cell death in response to oxidative stress	3
cellular response to vascular endothelial growth factor stimulus	3
cellular response to virus	3
defense response	3
enzyme linked receptor protein signaling pathway	3
mitotic cell cycle phase transition	3
negative regulation of cardiac muscle hypertrophy	3
negative regulation of catalytic activity	3
negative regulation of cation channel activity	3
negative regulation of cell communication	3
negative regulation of cell differentiation	3

negative regulation of cell migration involved in sprouting angiogenesis	3
negative regulation of cell population proliferation	3
negative regulation of ion transmembrane transporter activity	3
negative regulation of mitotic cell cycle	3
negative regulation of muscle cell differentiation	3
negative regulation of muscle hypertrophy	3
negative regulation of oxidative stress-induced cell death	3
negative regulation of oxidoreductase activity	3
negative regulation of potassium ion transmembrane transport	3
negative regulation of potassium ion transmembrane transporter activity	3
negative regulation of potassium ion transport	3
negative regulation of signal transduction	3
negative regulation of signaling	3
negative regulation of vascular associated smooth muscle cell proliferation	3
negative regulation of voltage-gated potassium channel activity	3
neuroinflammatory response	3
organonitrogen compound biosynthetic process	3
osteoblast differentiation	3
positive regulation of cell migration	3
positive regulation of cell motility	3
positive regulation of cellular component movement	3
positive regulation of developmental process	3
positive regulation of locomotion	3
positive regulation of myotube differentiation	3
programmed cell death	3
regulation of apoptotic process	3
regulation of cardiac muscle cell apoptotic process	3
regulation of cell death	3
regulation of cell differentiation	3
regulation of cellular protein metabolic process	3
regulation of mitotic cell cycle phase transition	3
regulation of myotube differentiation	3
regulation of neuroinflammatory response	3
regulation of oxidative stress-induced cell death	3
regulation of oxidoreductase activity	3
regulation of potassium ion transmembrane transporter activity	3
regulation of programmed cell death	3
regulation of striated muscle cell apoptotic process	3

regulation of tumor necrosis factor production	3
regulation of tumor necrosis factor superfamily cytokine production	3
striated muscle cell apoptotic process	3
tumor necrosis factor production	3
tumor necrosis factor superfamily cytokine production	3
cardiac muscle tissue regeneration	2
cell population proliferation	2
interleukin-17 production	2
interleukin-6 production	2
negative regulation of cation transmembrane transport	2
negative regulation of cell adhesion molecule production	2
negative regulation of cell death	2
negative regulation of cell migration	2
negative regulation of cell motility	2
negative regulation of cellular component movement	2
negative regulation of developmental process	2
negative regulation of ERK1 and ERK2 cascade	2
negative regulation of interleukin-17 production	2
negative regulation of interleukin-6 production	2
negative regulation of intracellular signal transduction	2
negative regulation of ion transmembrane transport	2
negative regulation of lipid metabolic process	2
negative regulation of locomotion	2
negative regulation of molecular function	2
negative regulation of NIK/NF-kappaB signaling	2
negative regulation of nitrogen compound metabolic process	2
negative regulation of osteoblast differentiation	2
negative regulation of osteoblast proliferation	2
negative regulation of protein metabolic process	2
negative regulation of response to external stimulus	2
negative regulation of transmembrane transport	2
negative regulation of tumor necrosis factor production	2
negative regulation of tumor necrosis factor superfamily cytokine production	2
osteoblast proliferation	2
positive regulation of blood vessel endothelial cell migration	2
positive regulation of cardiac muscle tissue	2

regeneration	
potassium ion transmembrane transport	2
protein kinase B signaling	2
regulation of cardiac muscle tissue regeneration	2
regulation of cell population proliferation	2
regulation of cellular response to growth factor stimulus	2
regulation of cellular response to vascular endothelial growth factor stimulus	2
regulation of defense response	2
regulation of inflammatory response	2
regulation of interleukin-17 production	2
regulation of interleukin-6 production	2
regulation of intracellular signal transduction	2
regulation of mitotic cell cycle	2
regulation of molecular function	2
regulation of nitrogen compound metabolic process	2
regulation of osteoblast differentiation	2
regulation of osteoblast proliferation	2
regulation of potassium ion transmembrane transport	2
regulation of potassium ion transport	2
regulation of protein kinase B signaling	2
regulation of response to external stimulus	2
regulation of vascular associated smooth muscle cell differentiation	2
regulation of vascular associated smooth muscle cell proliferation	2
regulation of vascular endothelial growth factor signaling pathway	2
tissue regeneration	2
vascular associated smooth muscle cell differentiation	2
vascular associated smooth muscle cell proliferation	2

Table S3: Imaging phenotype and corresponding image features utilized in this study with detailed references

Texture Features	GLCM (Gray-Level Co-Occurence Matrix):<br>- Autocorrelation (autoc)[1, 2]<br>- Contrast (contr) [1, 3]<br>- Correlation Matlab (corrm) [4]<br>- Correlation (corrp) [1, 3]<br>- Cluster Prominence (cprom) [3]

	- Cluster Shade (cshad) [3]<br>- Dissimilarity (dissi) [3]<br>- Energy matlab (energ) [1, 3]<br>- Entropy (entro) [3]<br>- Homogeneity from matlab (homom) [4]<br>- Homogeneity (homop) [3]<br>- Maximum probability (maxpr) [3]<br>- Sum of squares: Variance (sosvh) [1]<br>- Sum average (savgh) [1]<br>- Sum variance (svarh) [1]<br>- Sum entropy (senth) [1]<br>- Difference variance (dvarh) [1]<br>- Difference entropy (denth) [1]<br>- Information measure of correlation1 (inf1h) [1]<br>- Information measure of correlation2 (inf2h) [1]<br>- Inverse difference (INV) [4]<br>- Inverse difference normalized (INN) (indnc) [4]<br>- Inverse difference moment normalized (idmnc) [4]
Texture Features	GLSZM (Gray-Level Size Zone Matrix):<br>- Small Zone Emphasis (SZE) [2, 5]<br>- Large Zone Emphasis (LZE) [2, 5]<br>- Gray-Level Nonuniformity (GLN), adapted from Galloway and Thibault et al. [2, 5]<br>- Zone-Size Nonuniformity (ZSN), adapted from Galloway and Thibault et al. [2, 5]<br>- Zone Percentage (ZP), adapted from Galloway [2, 5]<br>- Low Gray-Level Zone Emphasis (LGZE) [5, 6]<br>- High Gray-Level Zone Emphasis (HGZE) [5, 6]<br>- Small Zone Low Gray-Level Emphasis (SZLGE) [5, 7]<br>- Small Zone High Gray-Level Emphasis (SZHGE) [5, 7]<br>- Large Zone Low Gray-Level Emphasis (LZLGE) [5, 7]<br>- Large Zone High Gray-Level Emphasis (LZHGE) [5, 7]<br>- Gray-Level Variance (GLV), adapted from Thibault et. al [5]<br>- Zone-Size Variance (ZSV), adapted from Thibault et. al [5]
Texture Features	GLRLM (Gray-Level Run-Length Matrix):<br>- Short Run Emphasis (SRE) [2]<br>- Long Run Emphasis (LRE) [2]<br>- Gray-Level Nonuniformity (GLN), adapted from Galloway [2]<br>- Run-Length Nonuniformity (RLN), adapted from Galloway [2]<br>- Run Percentage (RP), adapted from Galloway [2]<br>- Low Gray-Level Run Emphasis (LGRE) [6]<br>- High Gray-Level Run Emphasis (HGRE) [6]<br>- Short Run Low Gray-Level Emphasis (SRLGE) [7]<br>- Short Run High Gray-Level Emphasis (SRHGE) [7]<br>- Long Run Low Gray-Level Emphasis (LRLGE) [7]<br>- Long Run High Gray-Level Emphasis (LRHGE) [7]<br>- Gray-Level Variance (GLV), adapted from Thibault [5]

	- Run-Length Variance (RLV), adapted from Thibault [5]
Texture Features	NGTDM (Neighborhood Gray-Tone Difference Matrix):<br>- Coarseness [8]<br>- Contrast [8]<br>- Busyness [8]<br>- Complexity [8]<br>- Strength [8]
Shape	Geometric measurements:<br>- 3D Moments: [9]<br>o Moment1, Moment2, Moment3, Moment4, Moment5<br>- Compactness (Comp (unit)) [9]<br>- Compactness Discrete (CompDiscrete) [9]<br>- Eccentricity (eccentricity) from Matlab<br>- Ellongation Major Radius (Ell_MajRad) [9]<br>- Ellongation (Ell_Elon) [9]<br>- Ellongation Flatness (Ell_Flatness) [9]<br>- Fit Ellipse 3-dimensional fitting of an ellipse within the tumor volume (Spher (unit)) [10]<br>- Fitted volume for an ellipsoid (volEllipsoid (unit)) [9]<br>- RatioVolEllipsoid [9]
Morphology	- Density: [11]<br>o DCMin (unit), DCMax (unit), DCMean (unit), DCSD (unit)<br>- Integrated Density (IntDen) [11]<br>- Kurtosis from Matlab<br>- Min Grey Value (Min) [11]<br>- Max Grey Value (Max) [11]<br>- Mean Gray Value (Mean) [11]<br>- Skewness from Matlab<br>- Standard deviation Grey Value (Sigma) [11]<br>- Variance from Matlab
Size	- Volume (Vol (unit) from ImageJ) [9]<br>- volume from Matlab<br>- Feret (Feret (unit)) [9]<br>- Convex Hull (VolHull (unit)) [12]<br>- size from Matlab, computes the longest diameter<br>- Surface (Surf (unit)) [10]

Table S4: Summary of the data sets, features and acquisition methods

Dataset	Number of features	Method of acquisition
CT image features	86	Fiji, MATLAB, TCIA
Differentially expressed genes (DEGs)	7214	DEseq2, TCGA-LUAD Project
Differentially expressed miRNAs (DEMs)	147	DEseq2, TCGA-LUAD Project
GO Terms for DEGs	317	g:Profiler
GO Terms for DEMs	538	g:Profiler
Association of GO Terms for DEGs and image features	7634	Gene set enrichment analysis with Piano
Association of GO Terms for DEMs and image features	1156	Gene set enrichment analysis with Piano
Intersected GO Terms	11	Intersection of "Association of GO Terms for DEGs" and "Association of GO Terms for DEMs"

Table S5: Summary of software, libraries and methods used

Software	Version	Method
MATLAB	R2018b	Execute the Texture Toolbox
Fiji	8	Segmentation
3D-ROI Manager plugin for Fiji	3.94	3D intensity-, quantitative- and distance measurements
Texture Toolbox for MATLAB (https://github.com/mvallieres/radiomics/tree/master/TextureToolbox)	Commit id: 9a8c5cb	GLCM, GLRLM, GLSZM, NGTDM, Global
R statistical programming language	3.6.3	See R sessionInfo()
g:Profiler	Ensembl 102, Ensembl Genomes 49	Over-representation analysis
TFmiR2	The webserver was accessed on 15.06.2021	Gene regulatory network construction
Cytoscape	3.7.1	Visualization of TFmiR2 output
piano	2.20	Gene set analysis
stringr	1.4.0	Wrapper for processing tools for character text
psych	2.0.9	Functions for statistical analysis
ggplot2	3.3.5	Creating graphics
DESeq2	1.12.4	Identification of differentially expressed genes and miRNAs
reshape2	1.4.4	aggregation and restructuring of data
ComplexHeatmap	2.2.0	Visualization of Heatmaps
dplyr	1.0.7	Working with data frames
tidyr	1.1.4	Creation of tidy data for working with it
ggcorrplot	0.1.3	Visualizing correlation matrices
XML	3.99-0.3	Parsing XML files

org.Hs.eg.db	3.10.0	Annotation of Human genes
readr	1.4.0	reading csv files
vsn	3.54.0	Normalization of data
TCGAbiolinks	2.12.6	Facilitate access to TCGA data
data.table	1.13.0	Fast working with large data structures
SummarizedExperiment	1.14.1	Container structure

Figure S1 Visualization of the DEG data set for all GO terms from the piano analysis.

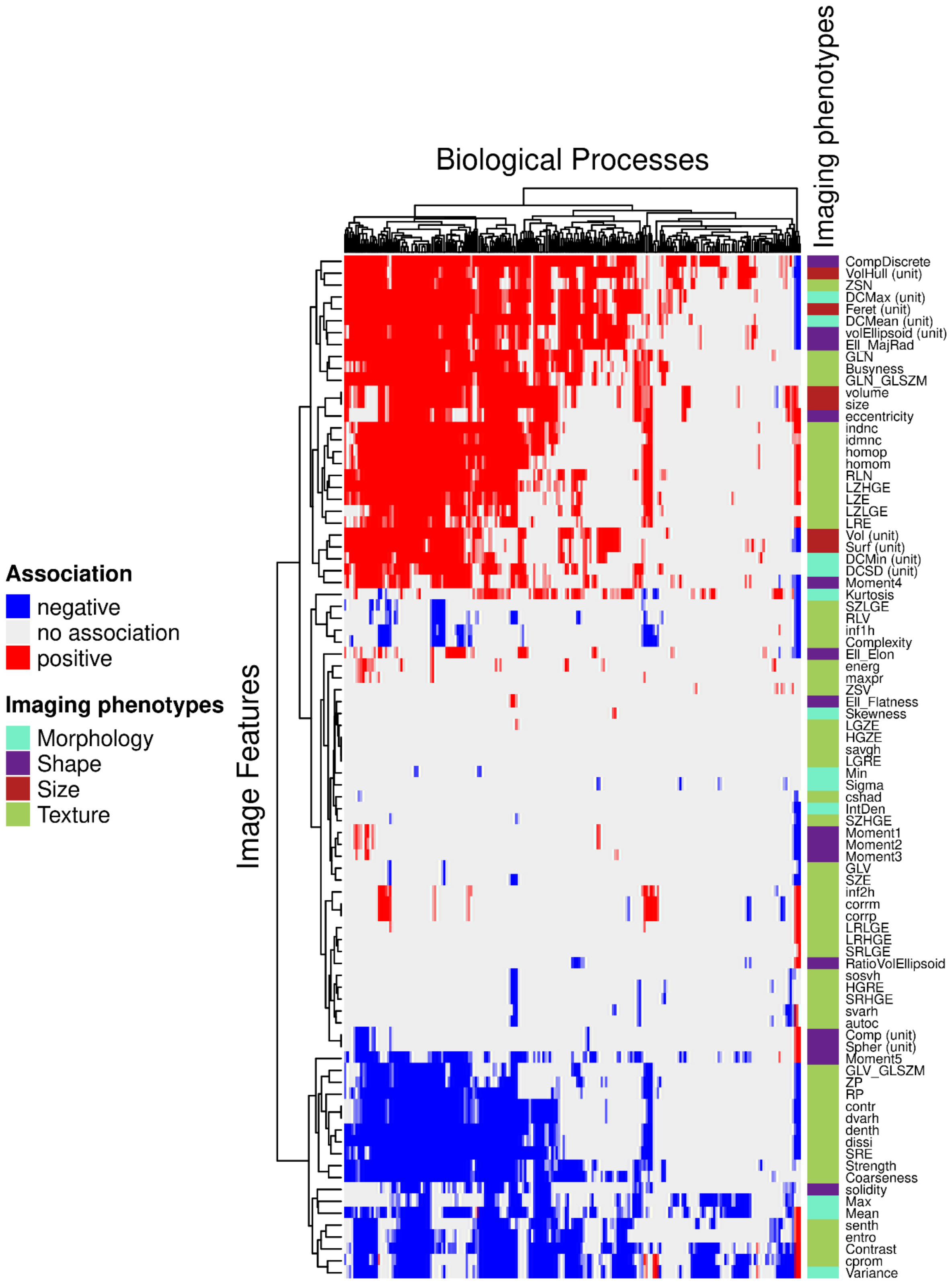

Figure S2 Visualization of the DEM data set for all GO terms from the piano analysis.

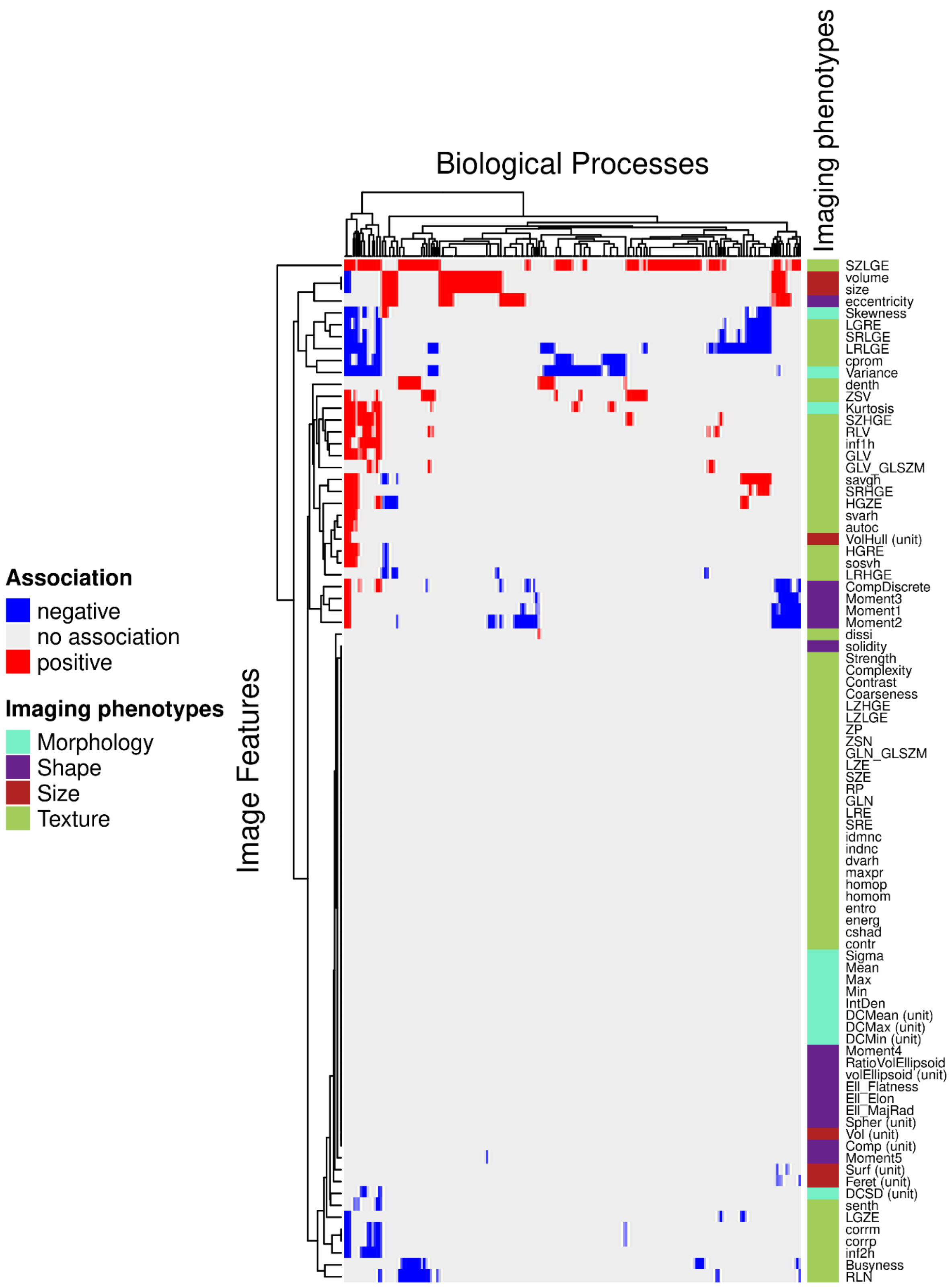

Figure S3 Spearman correlation coefficient (p<.05) between the DRF Variance and gene/miRNA expression within the term “regulation of signaling” of patient cluster 1. This figure is an extension of the corresponding RAM for “regulation of signaling”.

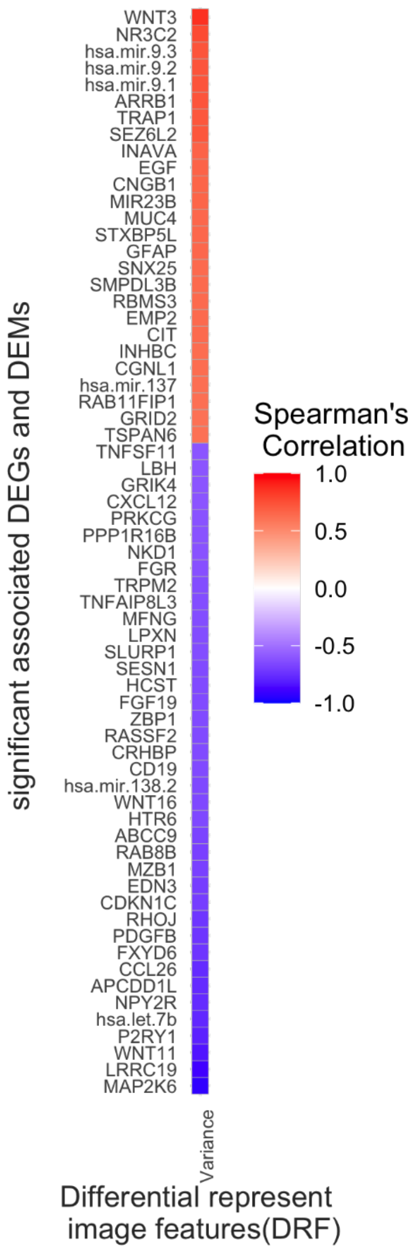

Figure S4 Spearman correlation coefficient (p<.05) between the DRF Variance and gene/miRNA expression within the term “regulation of signaling” of patient cluster 2. This figure is an extension of the corresponding RAM for “regulation of signaling”.

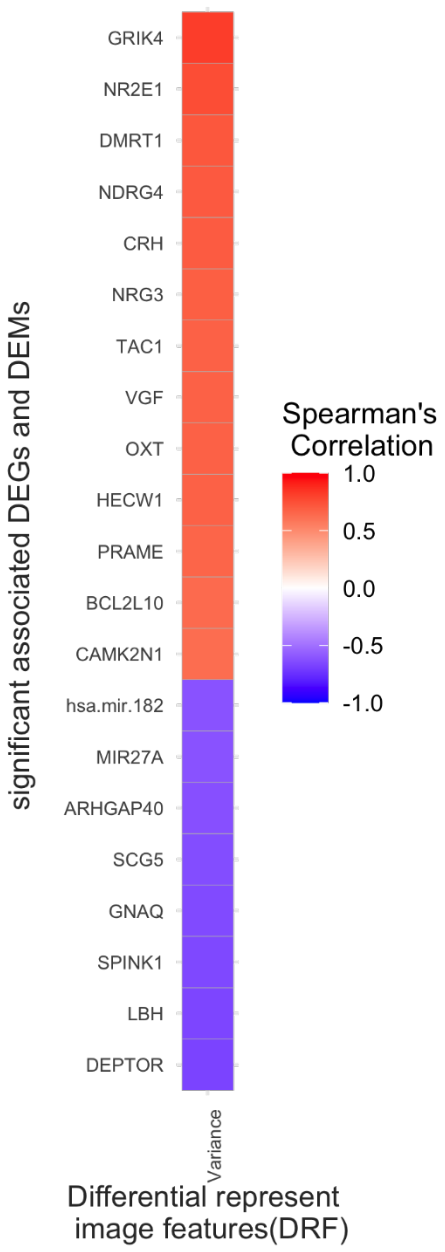

Figure S5 Spearman correlation coefficient (p<.05) between the DRFs and gene/miRNA expression within the term “cellular response to organic substance” of patient cluster 1. This figure is an extension of the corresponding RAM for “cellular response to organic substance”.

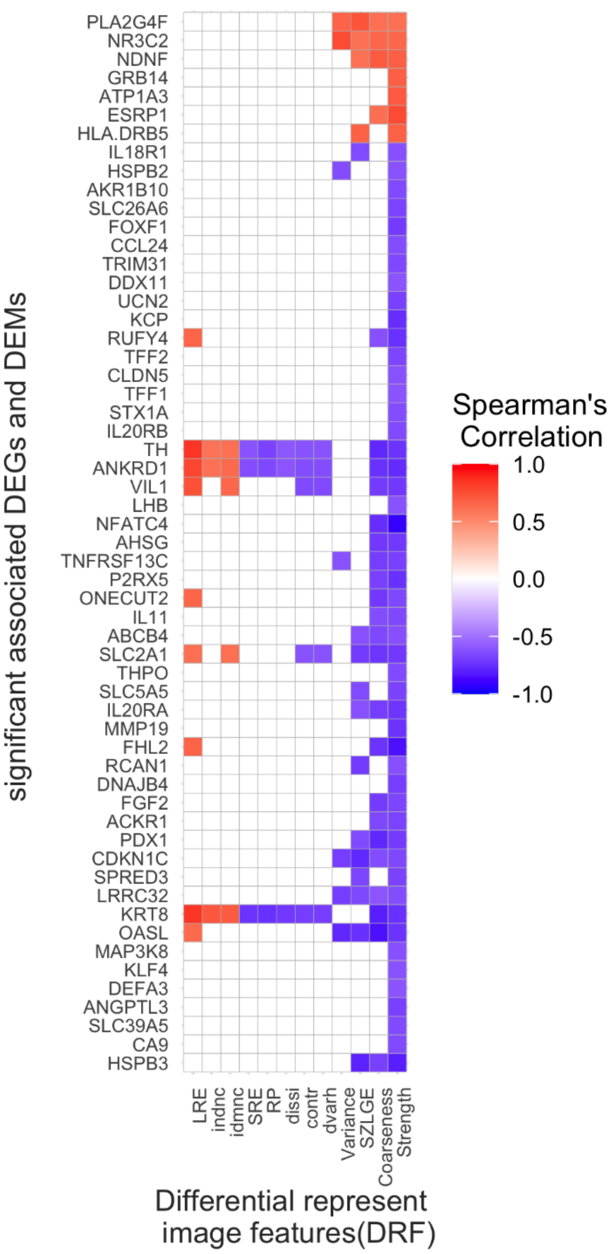

Figure S6 Spearman correlation coefficient (p<.05) between the DRFs and gene/miRNA expression within the term “cellular response to organic substance” of patient cluster 2. This figure is an extension of the corresponding RAM for “cellular response to organic substance”.

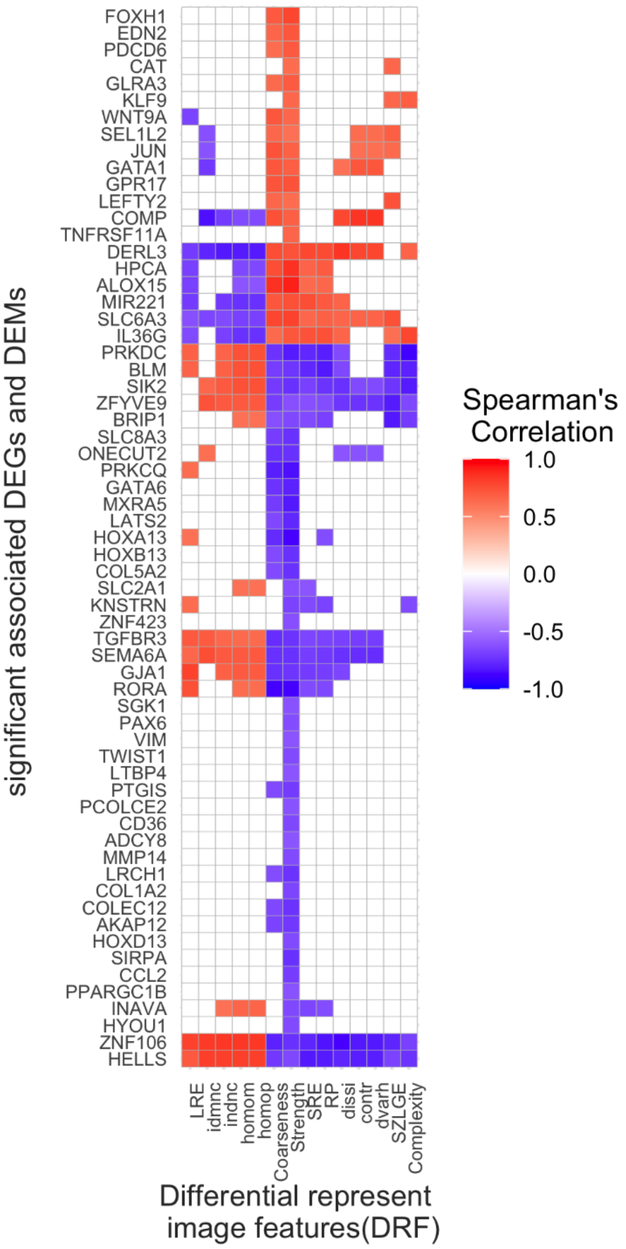

Radiogenomic association maps based on the overrepresentation analysis of the GO term's mRNA and miRNA expression

Figure S7 RAM for cell population proliferation based on mRNA expression clustering

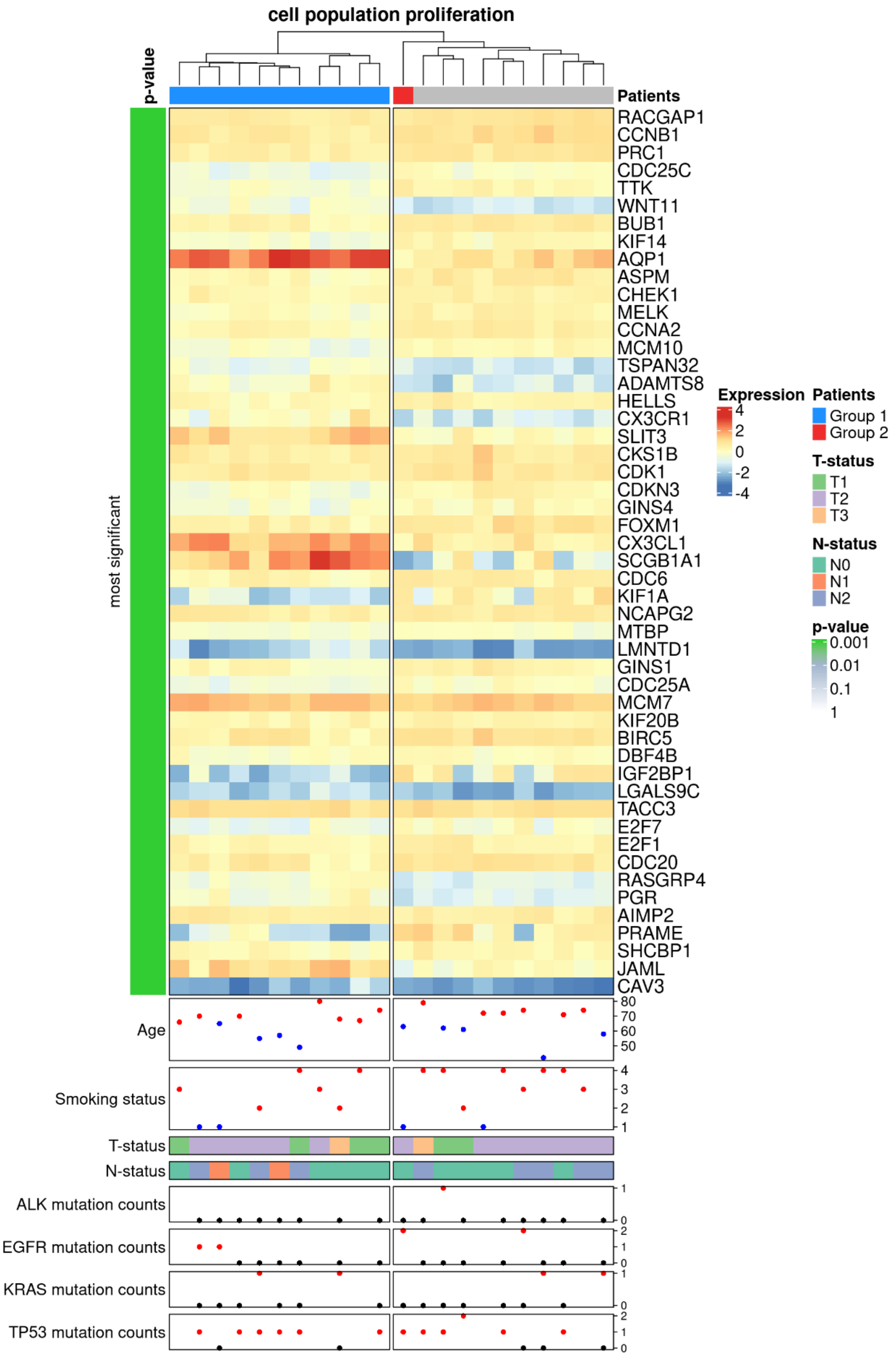

Figure S8 RAM for cell population proliferation based on miRNA expression clustering

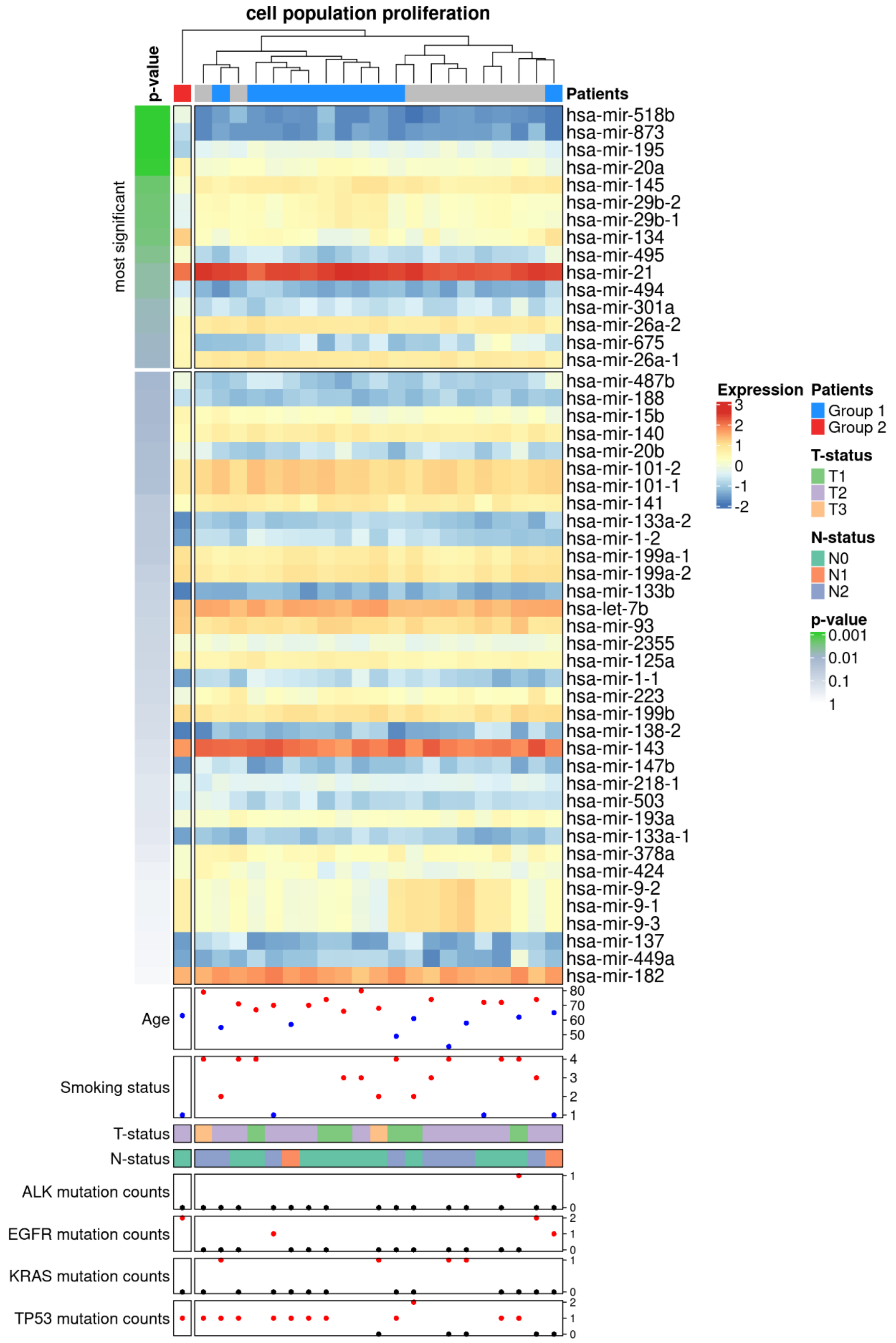

Figure S9 RAM for cellular response to chemical stimulus based on mRNA expression clustering

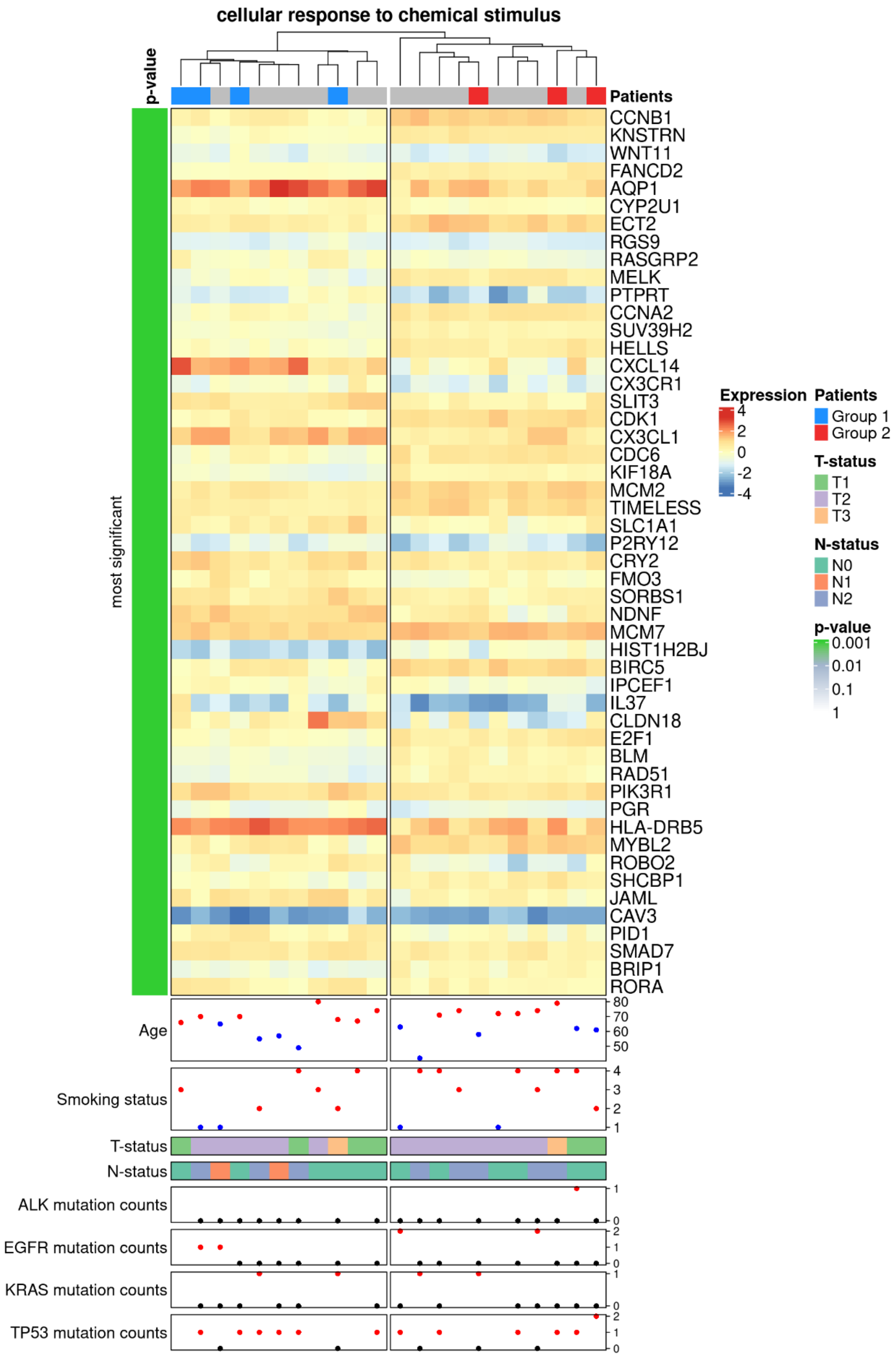

Figure S10 RAM for cellular response to chemical stimulus based on miRNA expression clustering

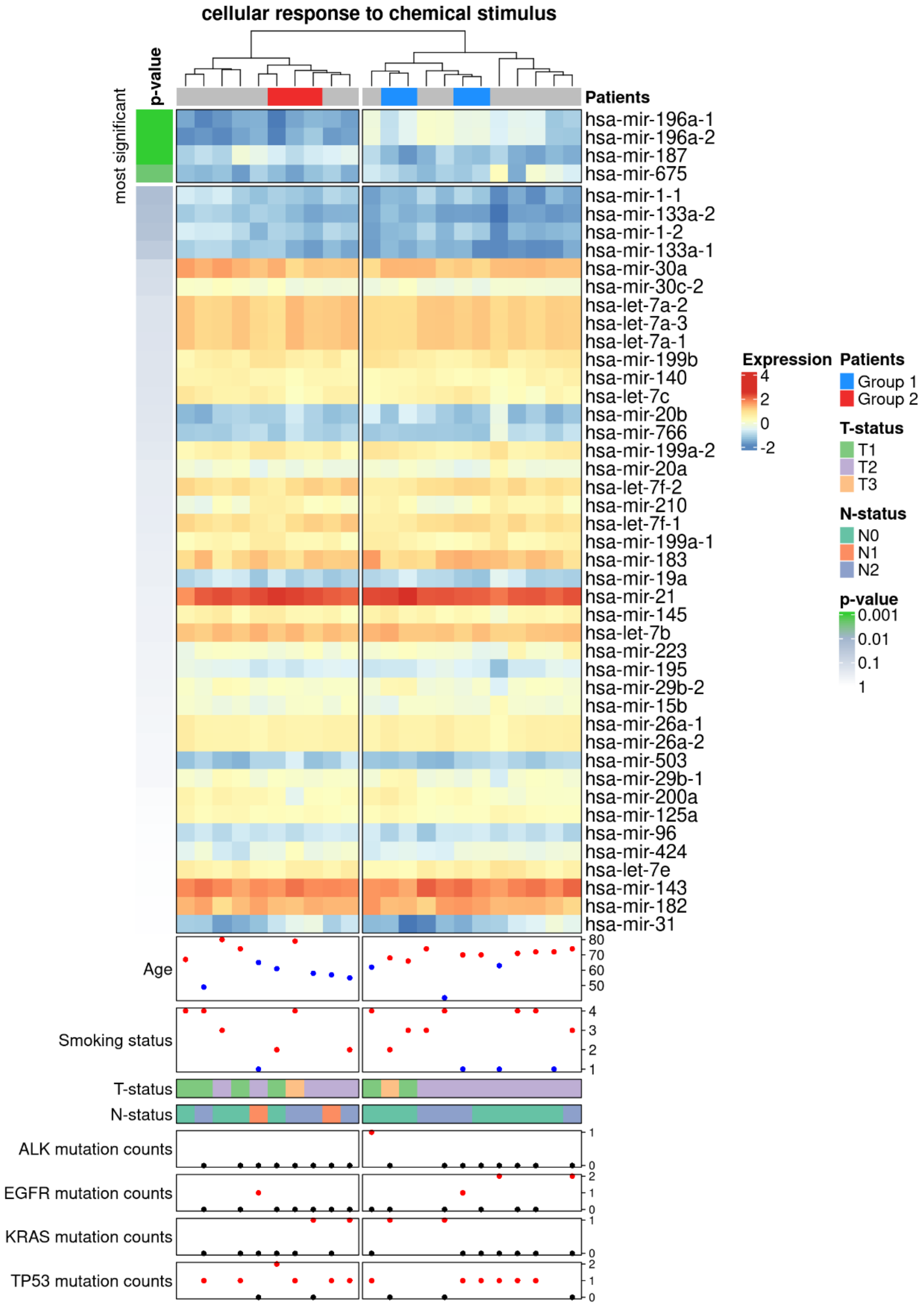

Figure S11 RAM for muscle structure development based on mRNA expression clustering

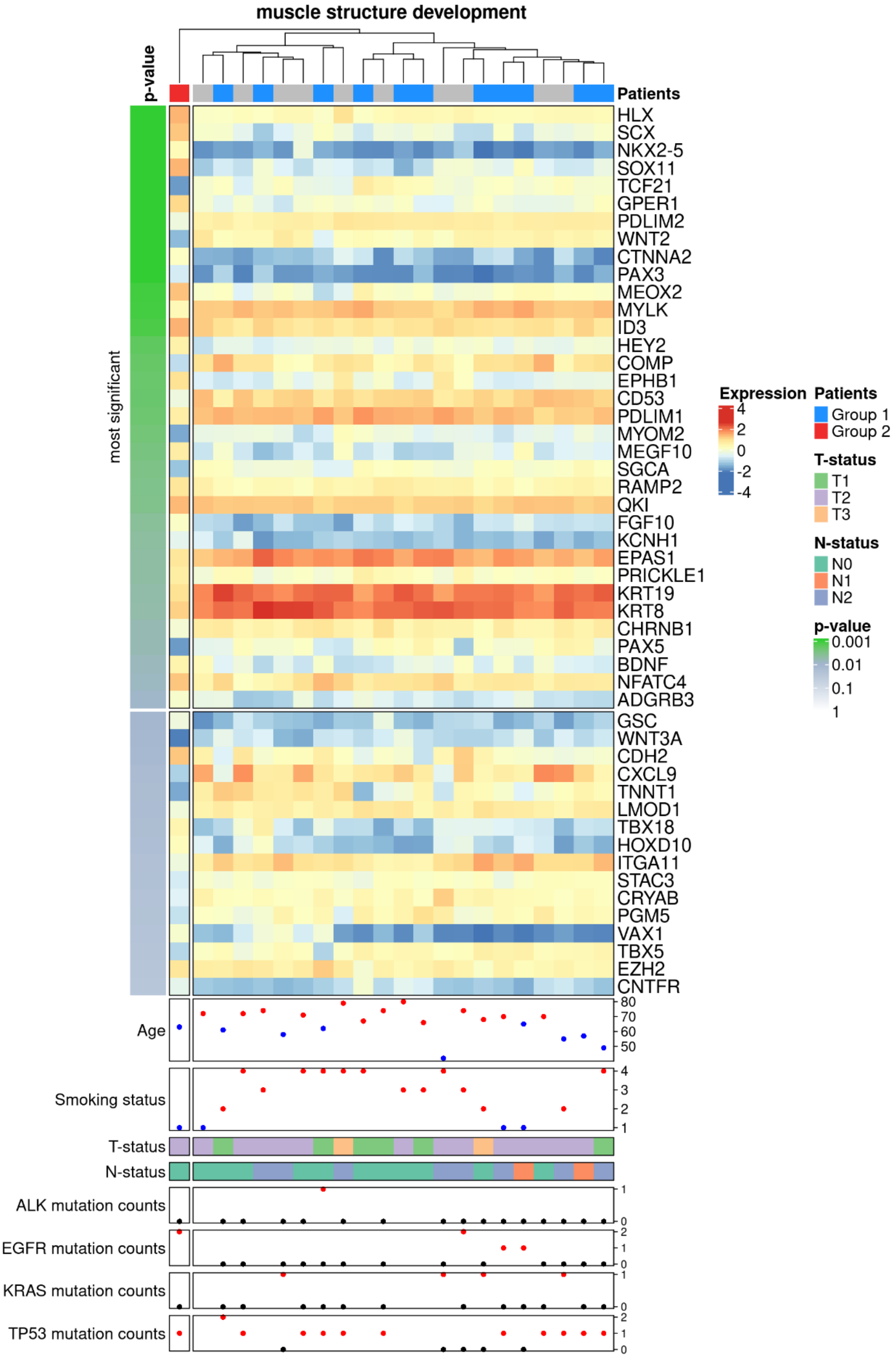

Figure S12 RAM for muscle structure development based on miRNA expression clustering

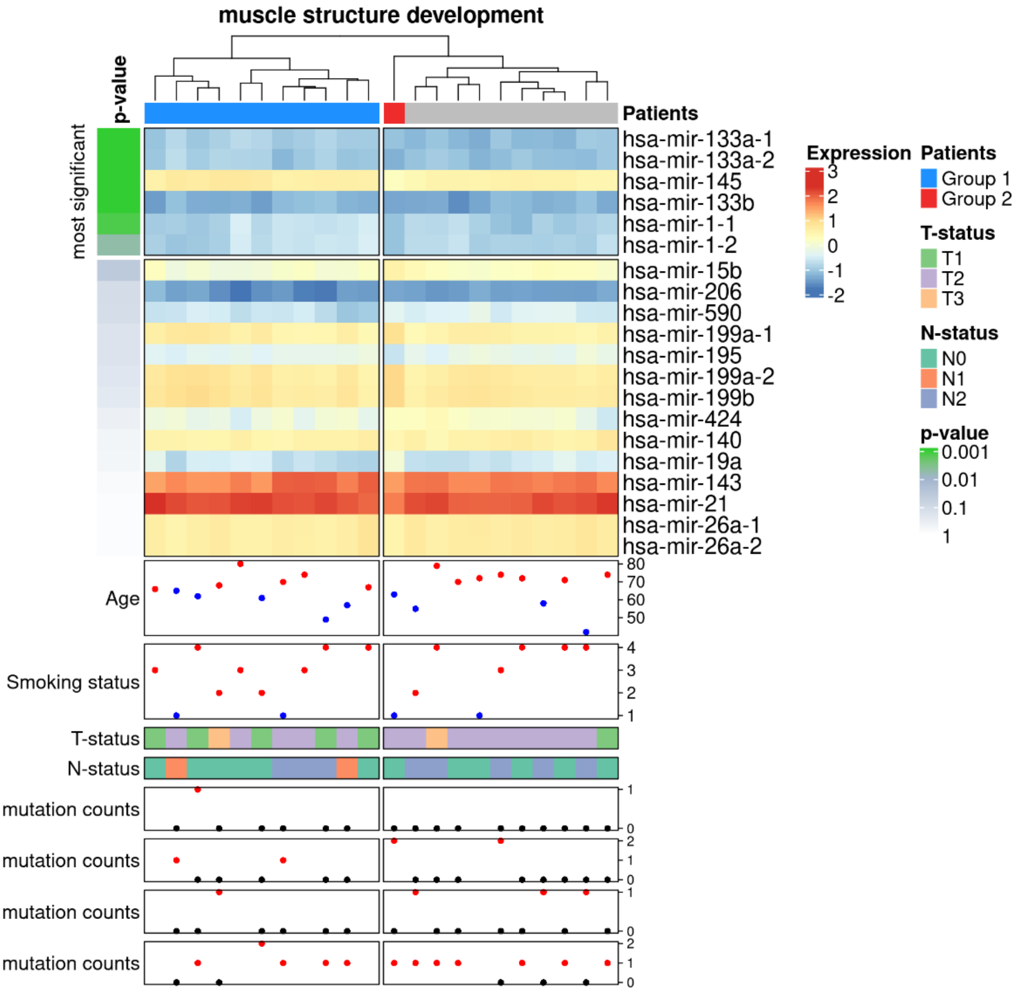

Figure S13 RAM for negative regulation of developmental process based on mRNA expression clustering

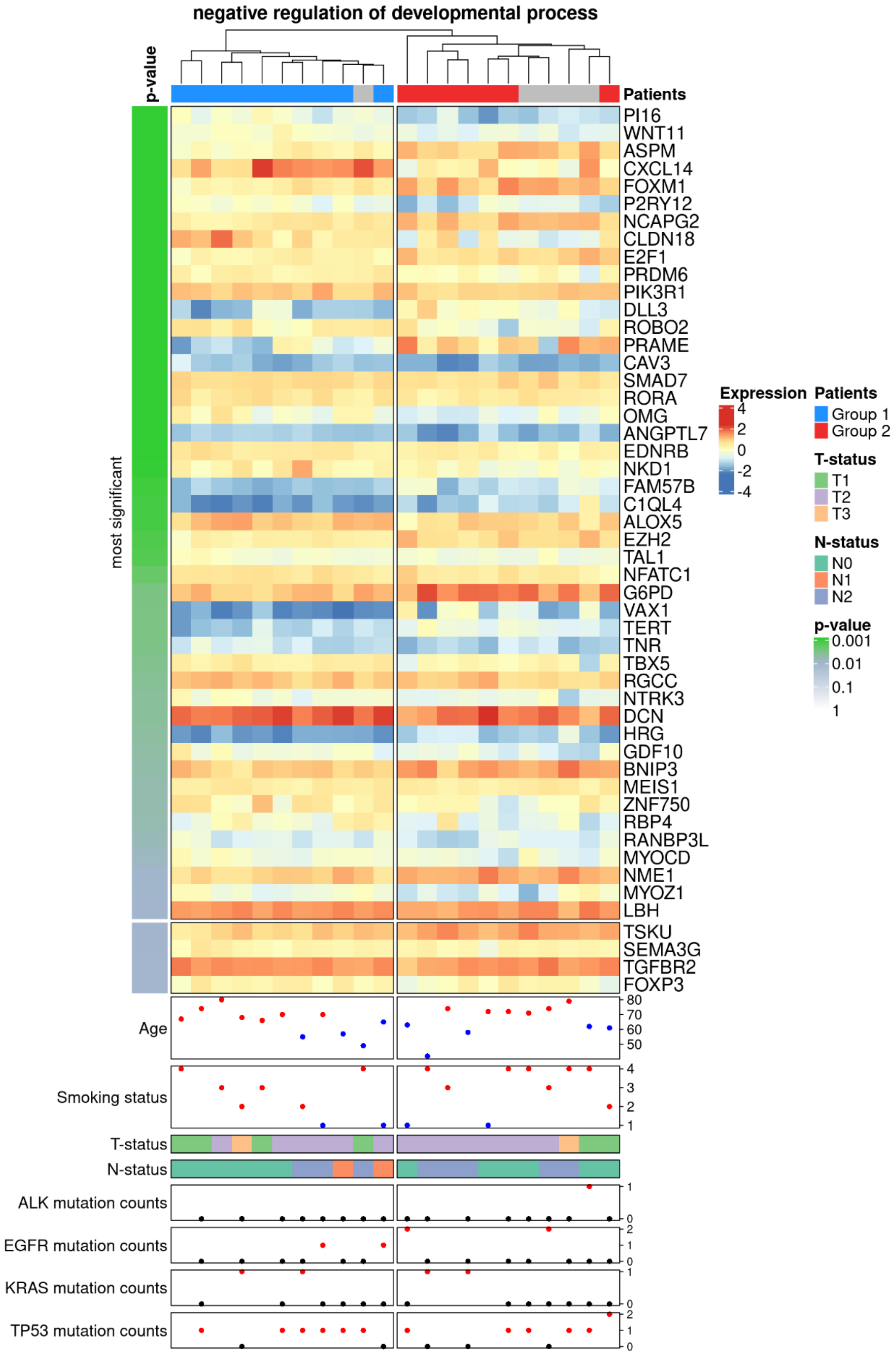

Figure S14 RAM for negative regulation of developmental process based on miRNA expression clustering

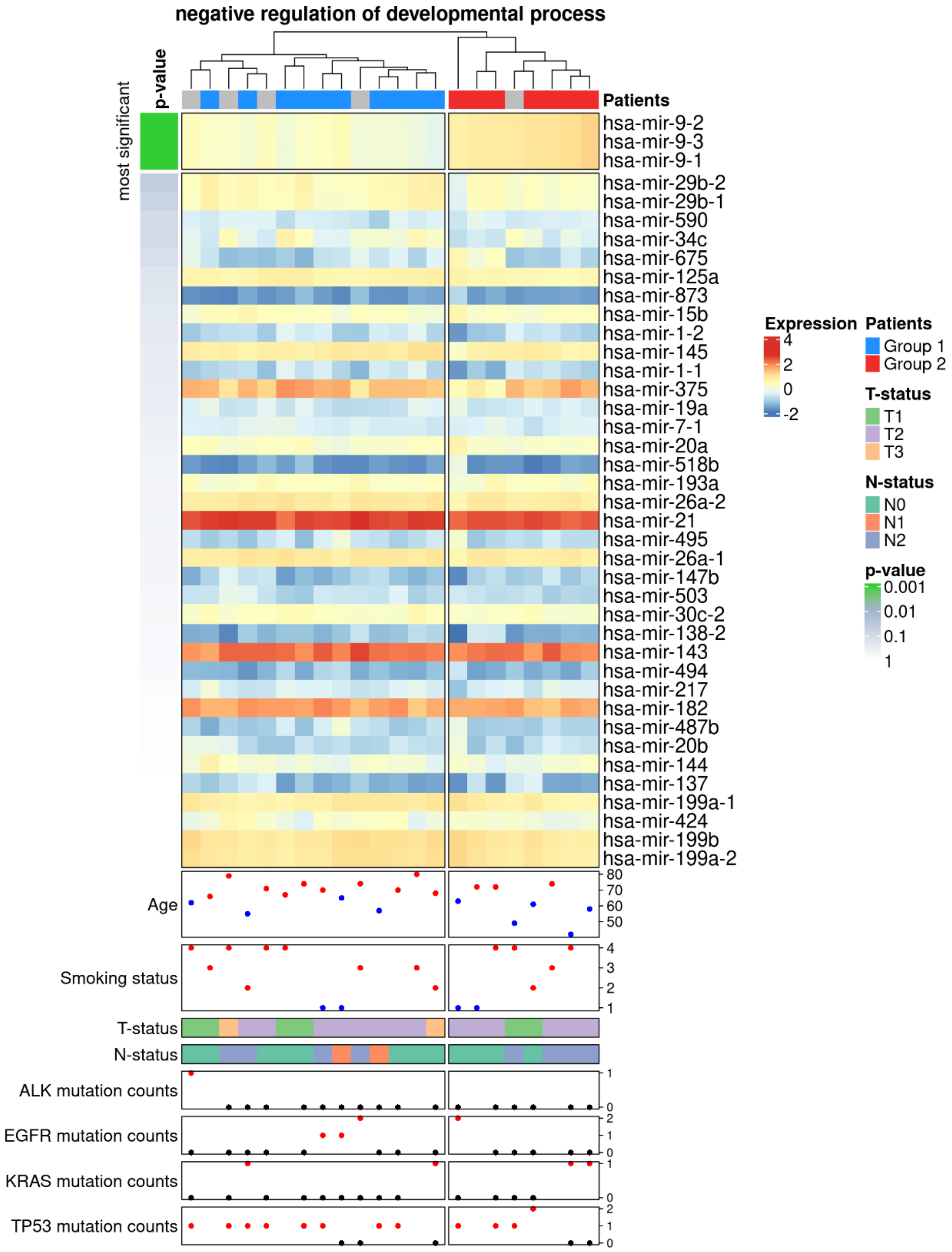

Figure 15 RAM for positive regulation of developmental process based on mRNA expression clustering

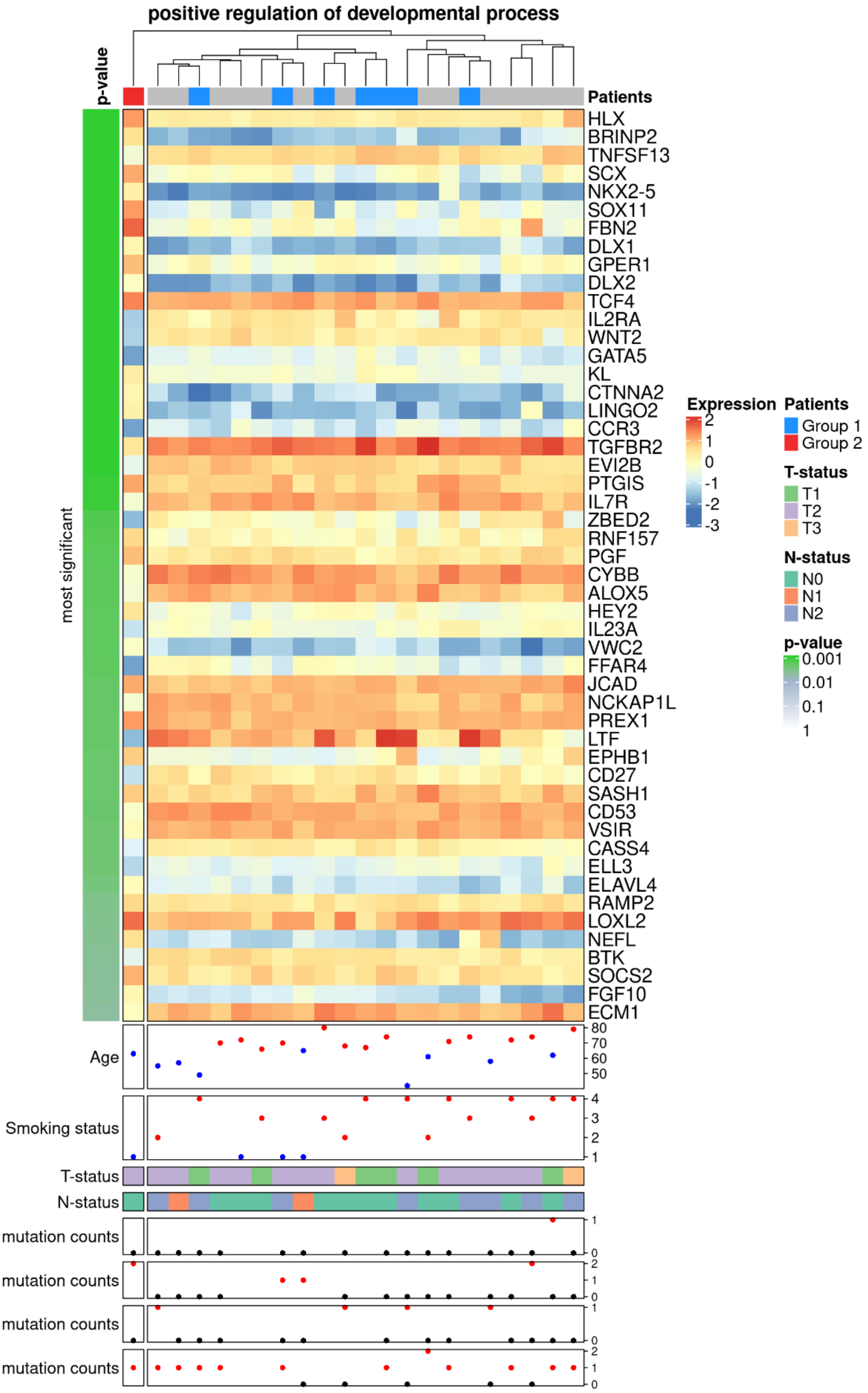

Figure S16 RAM for positive regulation of developmental process based on miRNA expression clustering

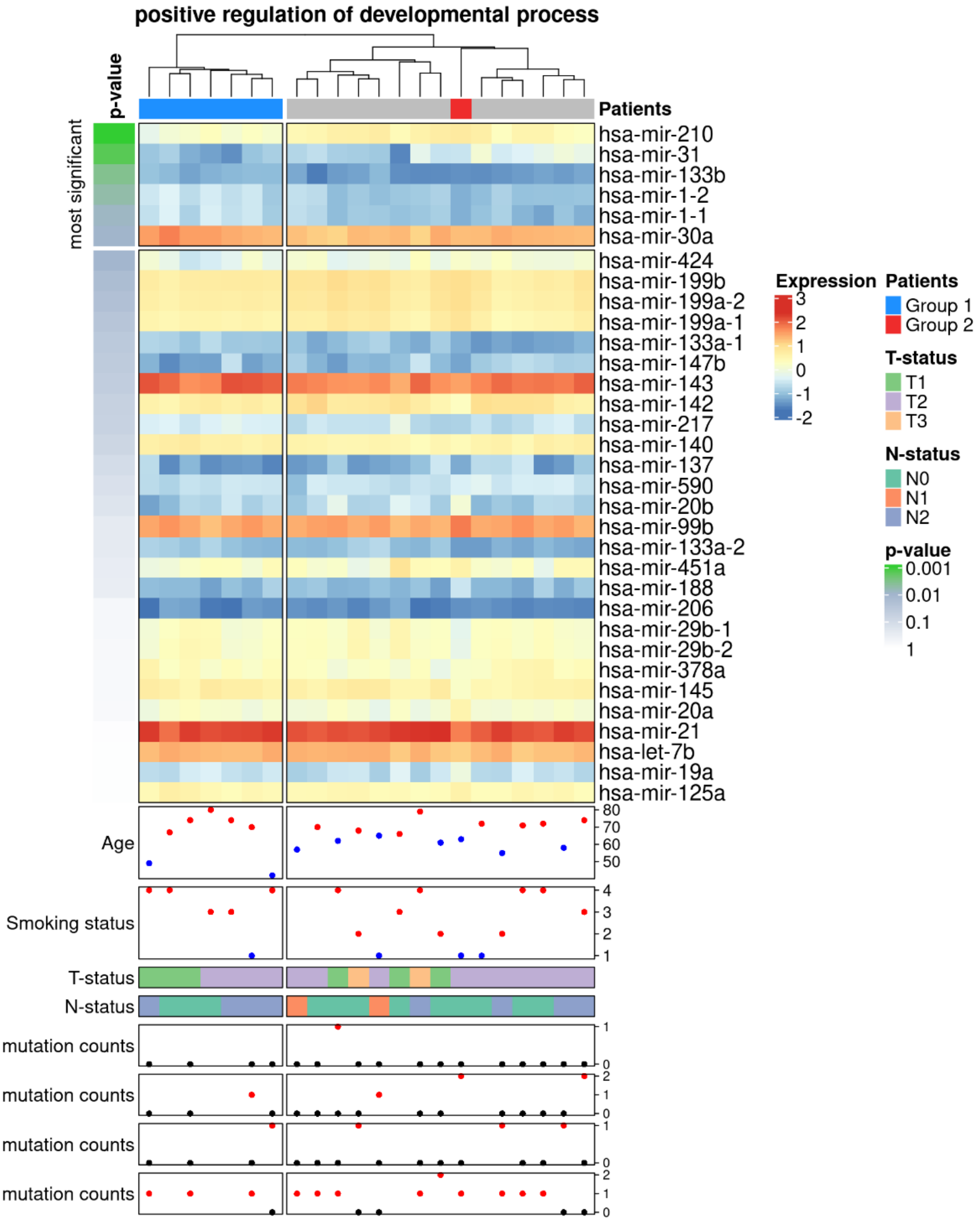

Figure 17 RAM for potassium ion transmembrane transport based on mRNA expression clustering

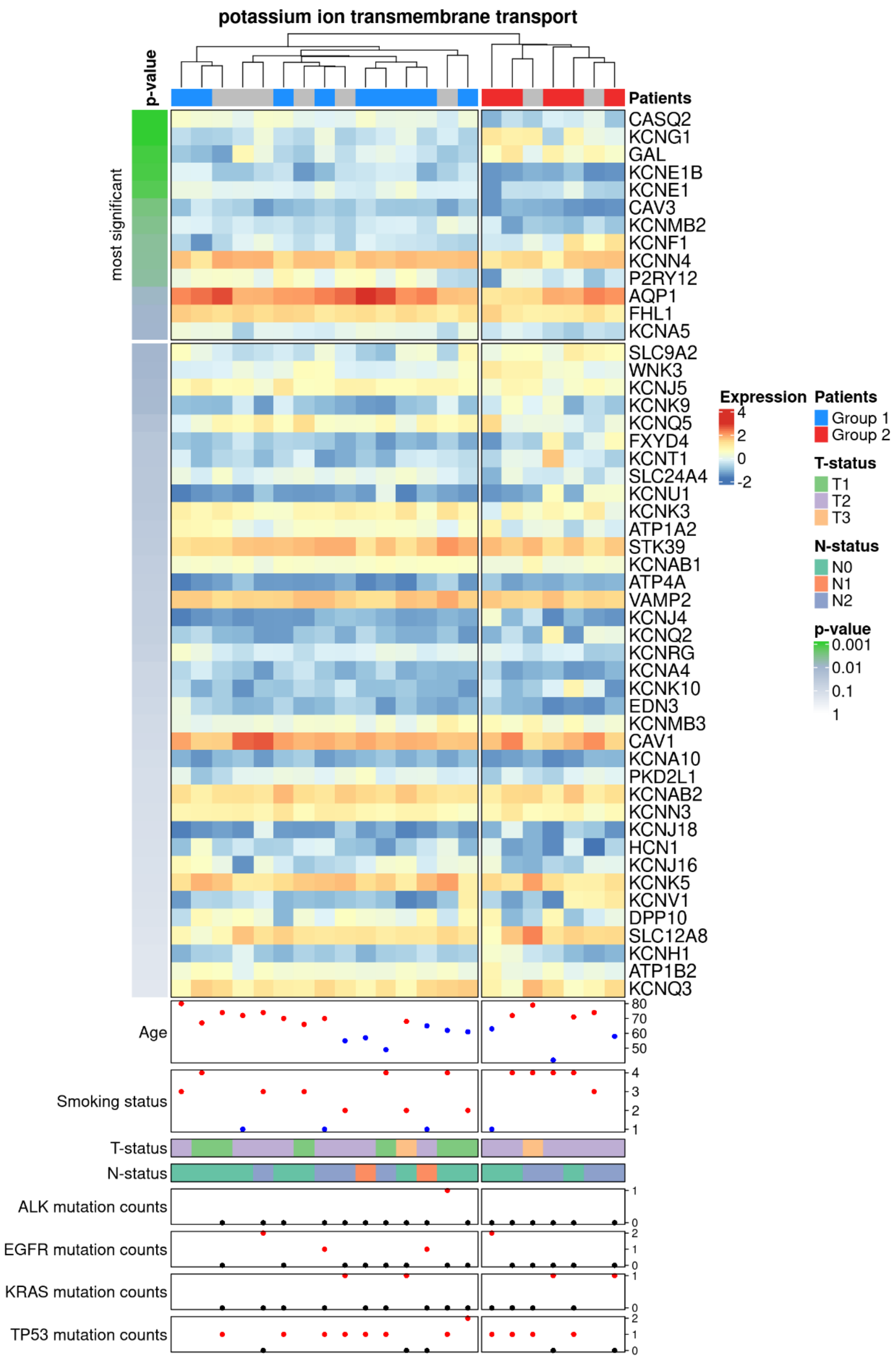

Figure S18 RAM for potassium ion transmembrane transport based on miRNA expression clustering

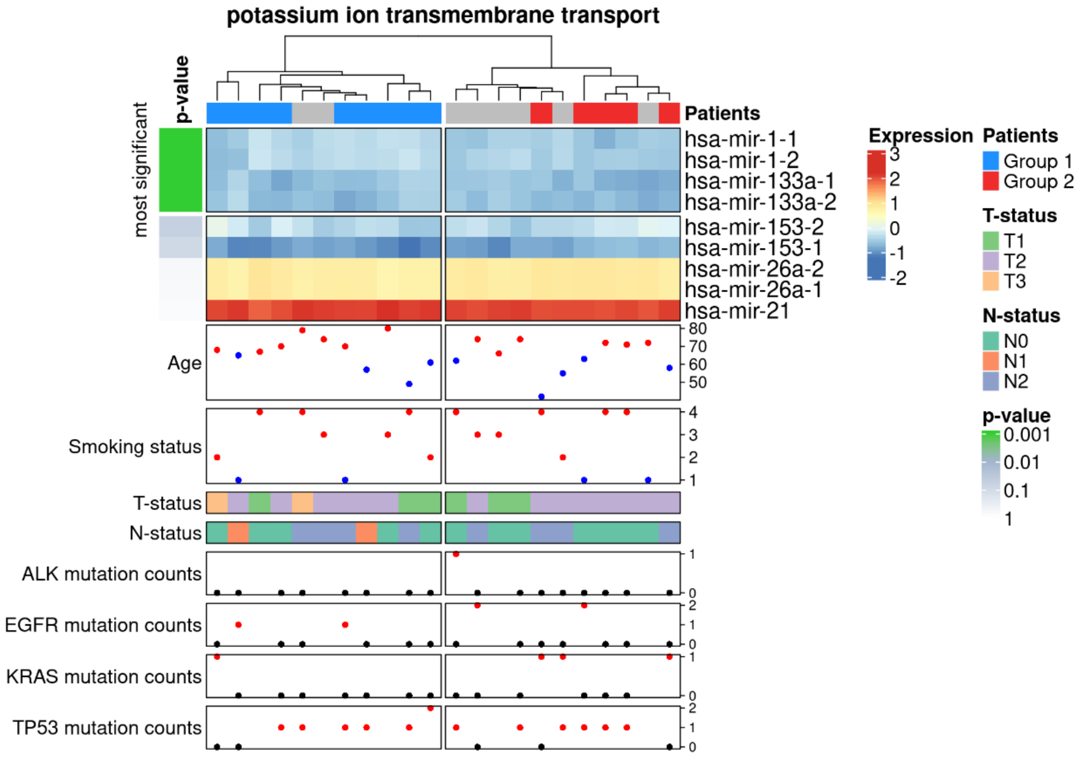

Figure S19 RAM for regulation of cell differentiation based on mRNA expression clustering

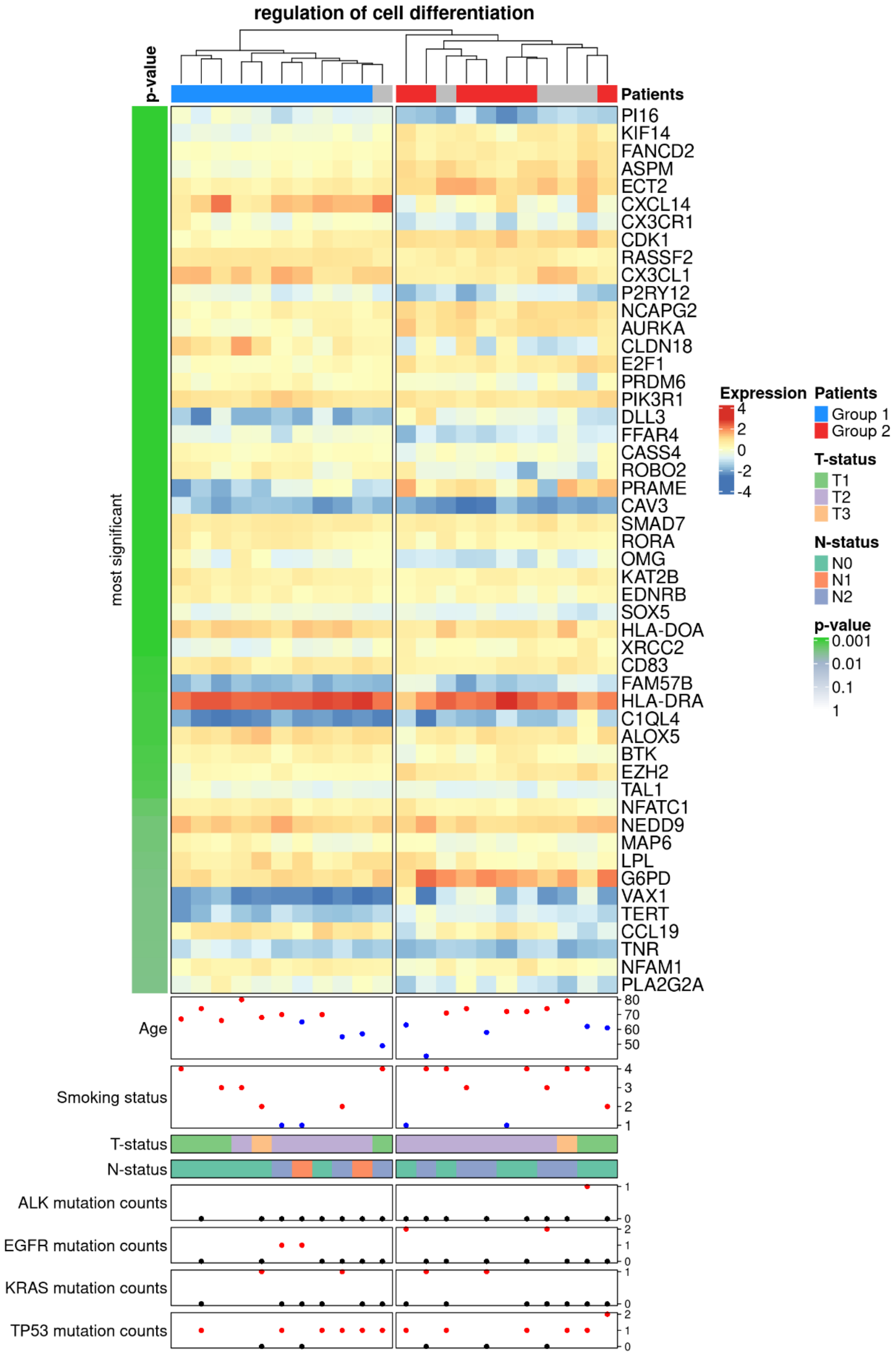

Figure S20 RAM for regulation of cell differentiation based on miRNA expression clustering

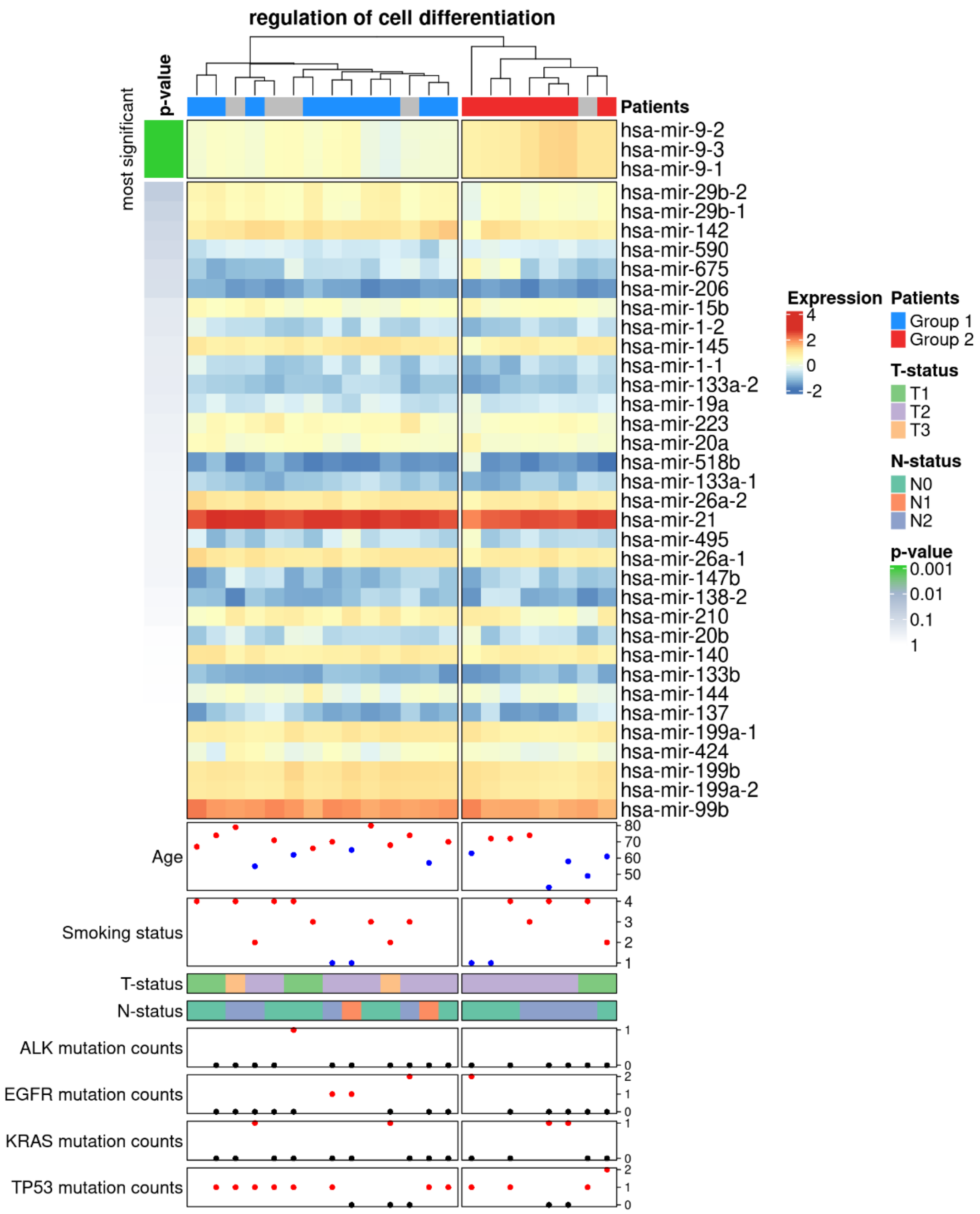

Figure S21 RAM for regulation of cell population proliferation based on mRNA expression clustering

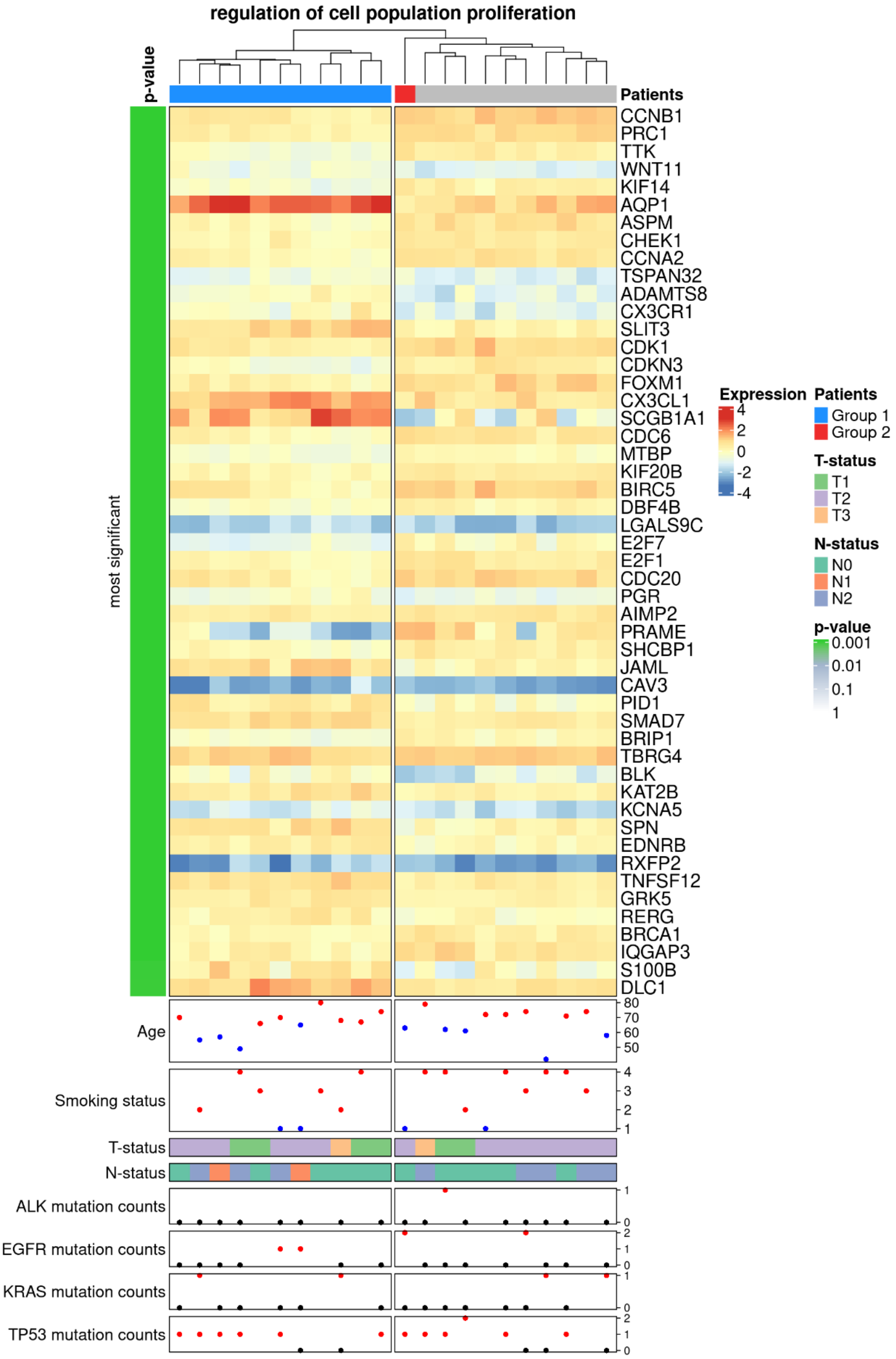

Figure S22 RAM for regulation of cell population proliferation based on miRNA expression clustering

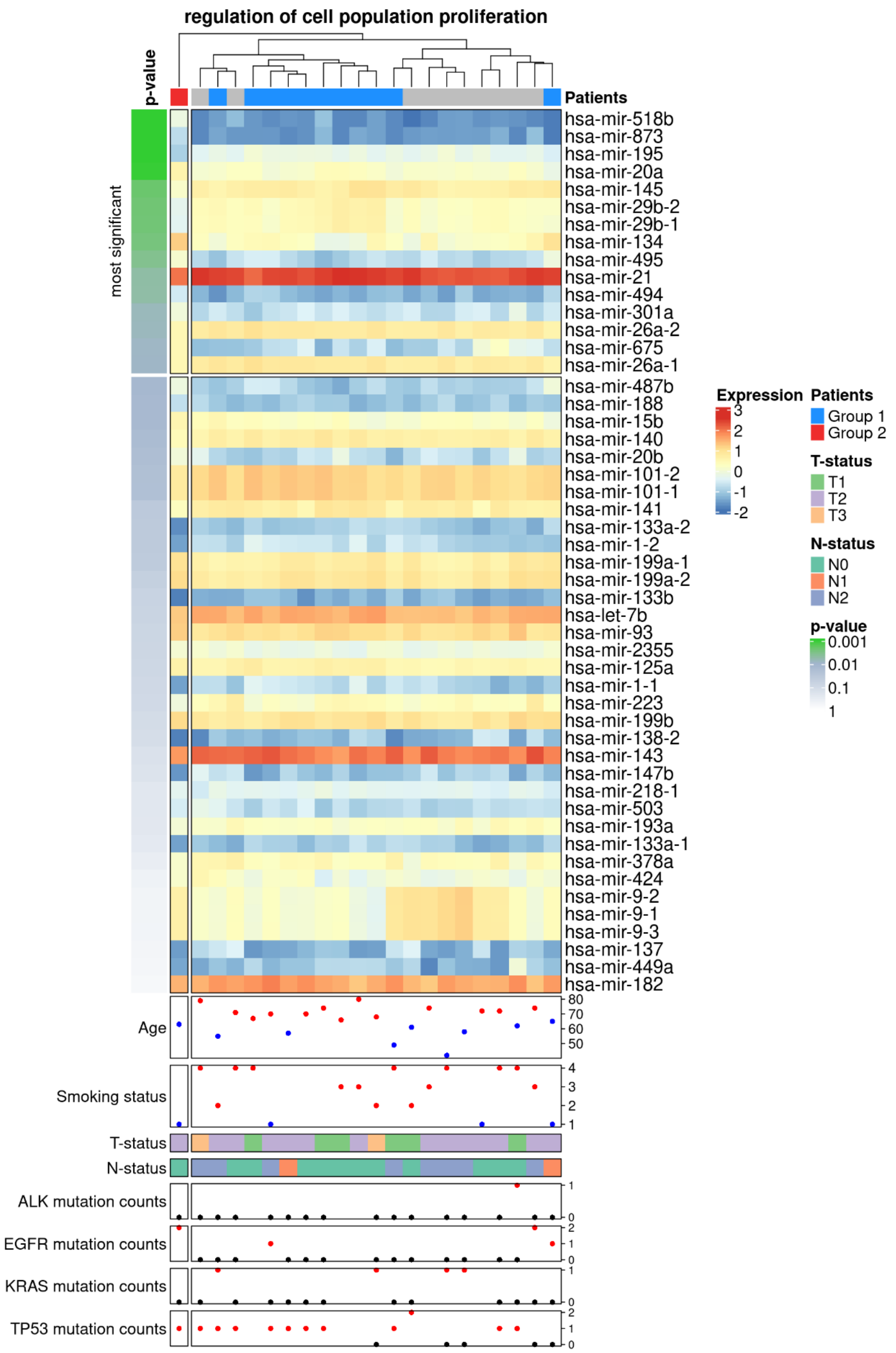

Figure S23 RAM for response to organic substance based on mRNA expression clustering

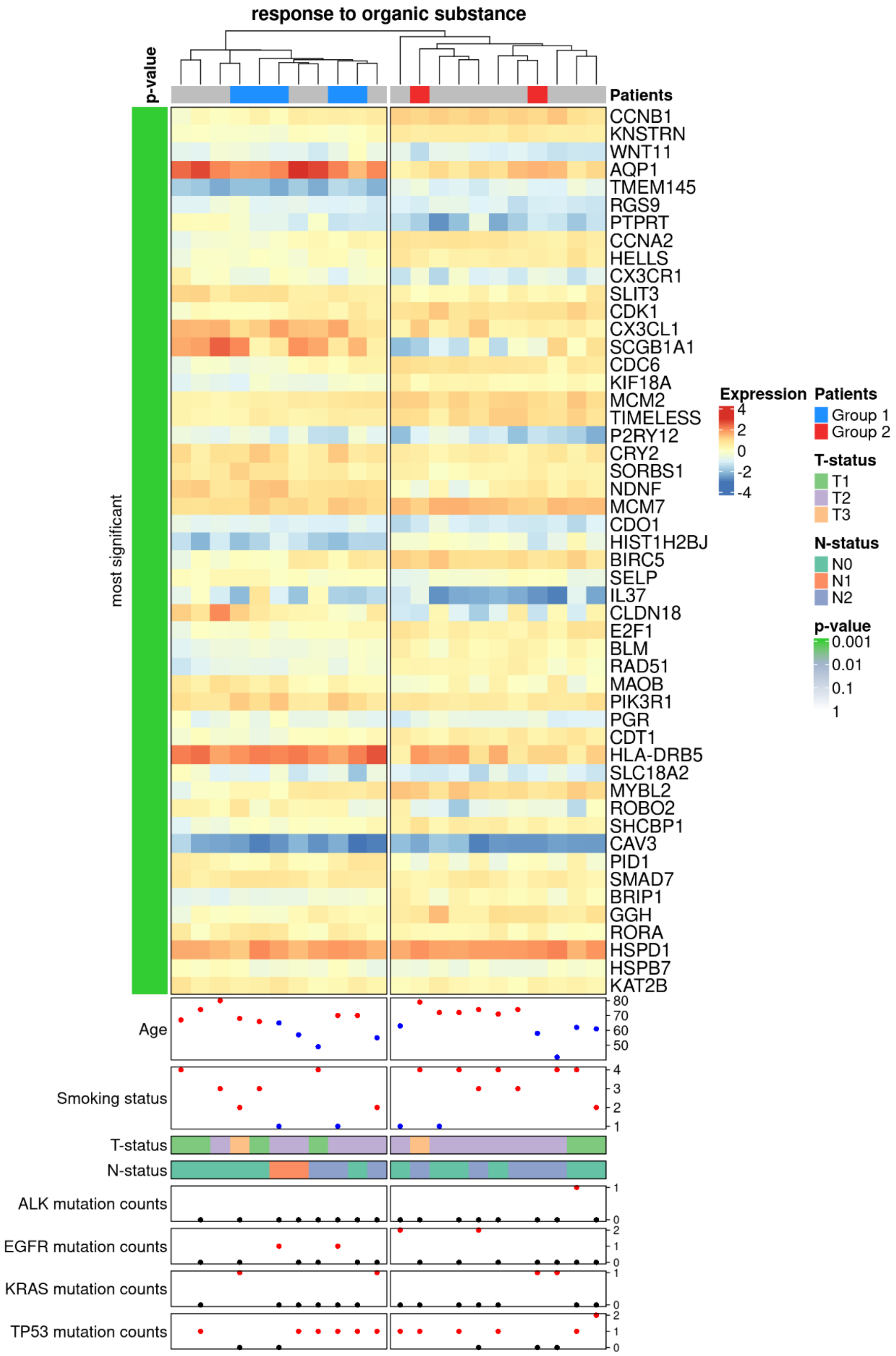

Figure S24 RAM for response to organic substance based on miRNA expression clustering

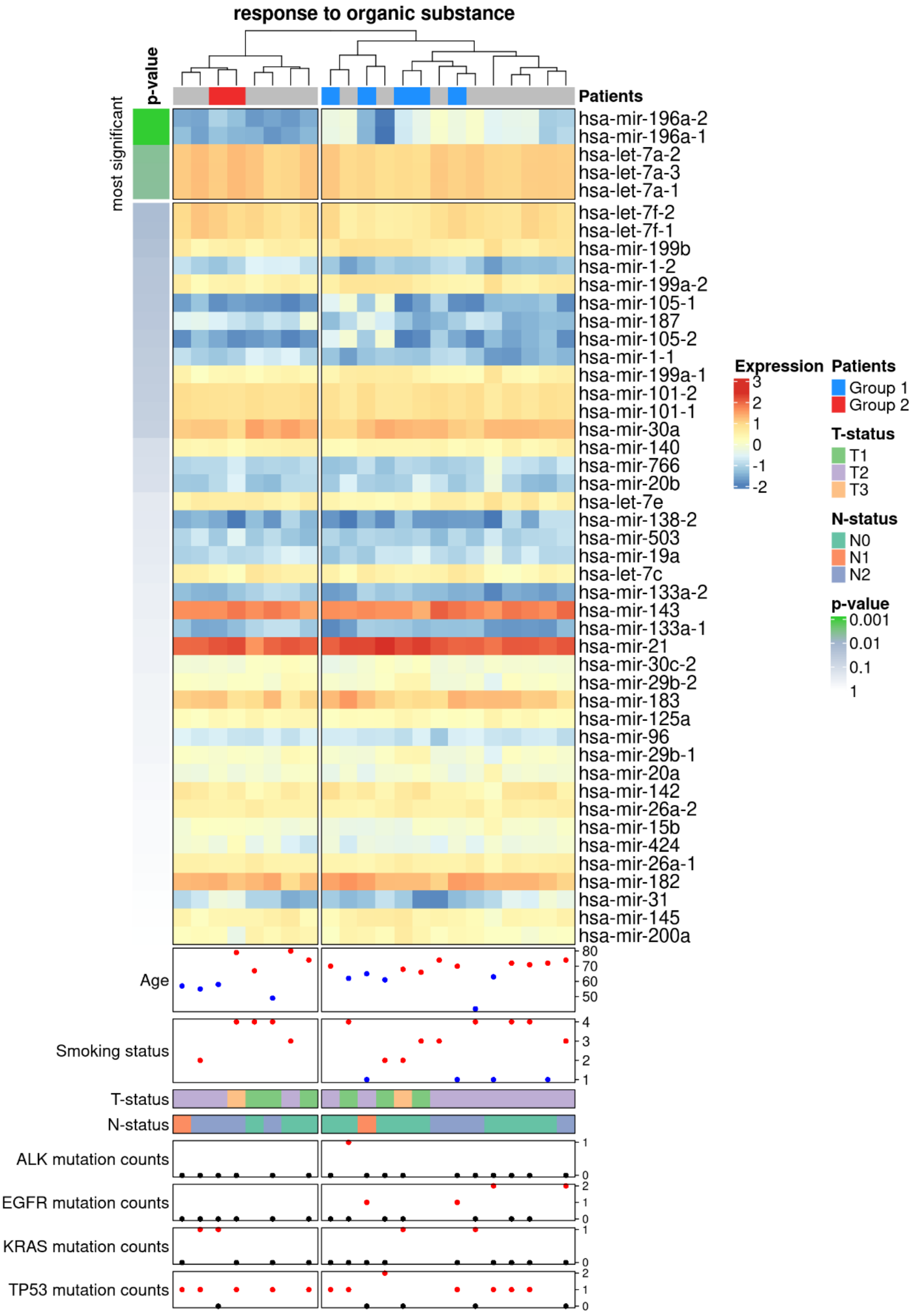

List of references

1. Robert M. Haralick, K. Sam Shanmugam, Its'hak Dinstein. Textural Features for Image Classification. IEEE Trans Syst Man, Cybern. 1973;3:610–21.

2. Galloway MM. Texture analysis using gray level run lengths. Comput Graph Image Process. 1975;4:172–9.

3. Soh L-K, Tsatsoulis C. Texture analysis of SAR sea ice imagery using gray level co-occurrence matrices. IEEE Trans Geosci Remote Sens. 1999;37:780–95.

4. Clausi DA. An analysis of co-occurrence texture statistics as a function of grey level quantization. Can J Remote Sens. 2002;28:45–62.

5. Thibault G, Fertil B, Navarro C, Pereira S, Cau P, Levy N, et al. Texture Indexes and Gray Level Size Zone Matrix Application to Cell Nuclei Classification. In: Pattern Recognition and Information Processing. 2009.

6. Chu A, Sehgal CM, Greenleaf JF. Use of gray value distribution of run lengths for texture analysis. Pattern Recognit Lett. 1990;11:415–9.

7. Dasarathy B V, Holder EB. Image characterizations based on joint gray level—run length distributions. Pattern Recognit Lett. 1991;12:497–502.

8. Amadasun M, King R. Textural features corresponding to textural properties. IEEE Trans Syst Man Cybern. 1989;19:1264–74.

9. Ollion J, Cochennec J, Loll F, Escudé C, Boudier T. TANGO: a generic tool for high-throughput 3D image analysis for studying nuclear organization. Bioinformatics. 2013;29:1840–1. doi:10.1093/bioinformatics/btt276.

10. Li Q, Griffiths JG. Least squares ellipsoid specific fitting. In: Geometric modeling and processing, 2004. proceedings. 2004. p. 335–40.

11. Andrey P, Kiêu K, Kress C, Lehmann G, Tirichine L, Liu Z, et al. Statistical analysis of 3D images detects regular spatial distributions of centromeres and chromocenters in animal and plant nuclei. PLoS Comput Biol. 2010;6.

12. Sheets KG, Bokkyoo J, Zhou Y, Winkler J, Zhu M, Petasis N, Gordon WC, Bazan NG. Topical Neuroprotectin D1 Attenuates Experimental CNV and Induces Activated Microglia Redistribution: ARVO Meeting Abstracts April 22, 2011. 2011.
